# Sulfane sulfur‐activated actinorhodin production and sporulation is maintained by a natural gene circuit in *Streptomyces coelicolor*

**DOI:** 10.1111/1751-7915.13637

**Published:** 2020-08-09

**Authors:** Ting Lu, Qun Cao, Xiuhua Pang, Yongzhen Xia, Luying Xun, Huaiwei Liu

**Affiliations:** ^1^ State Key Laboratory of Microbial Technology Shandong University Qingdao 266200 China; ^2^ School of Molecular Biosciences Washington State University Pullman WA 991647520 USA

## Abstract

Sulfane sulfur, including polysulfide and persulfide, is a newly identified cellular component present in microorganisms; however, its physiological functions are unclear. *Streptomyces coelicolor* M145 is a model strain of actinomycetes, which produces several polyketides, including actinorhodin. Herein, we found that both exogenously added and endogenously generated sulfane sulfur increased the actinorhodin production and accelerated spore formation of *S. coelicolor* M145. This bacterial species carries a natural gene circuit containing four genes that encode a CsoR‐like transcription factor (ScCsoR), persulfide dioxygenase (ScPDO), rhodanese and a sulfite transporter, which were shown to be responsible for sensing and removal of excessive sulfane sulfur. ScCsoR was observed to bind to the promoters of the four genes, thus repressing their transcription. Sulfane sulfur modified Cys37 of ScCsoR, and the modified ScCSoR did not bind to the promoters, thereby activating the transcription of ScPDO. The deletion of ScCsoR decreased cellular sulfane sulfur, while the deletion of ScPDO increased its levels. The increased sulfane sulfur promoted actinorhodin production and sporulation. This study unveiled a natural gene circuit for maintaining sulfane sulfur homeostasis in bacteria. Further, we identified the trigger effect of sulfane sulfur on actinorhodin production, presenting a new approach for activating polyketide gene clusters in actinomycetes.

## Graphical abstract

Sulfane sulfur activates actinorhodin production and sporulation in *Streptomyces coelicolor*. A gene cluster controlling intracellular sulfane sulfur levels is identified. Genetically modifying this gene cluster can change sulfane sulfur levels, thereby altering the actinorhodin production and sporulation.

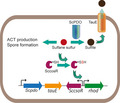

## Introduction

Sulfane sulfur, presenting as persulfide (HSSH and RSSH) and polysulfide (HSS*_n_*H, S*_n_*, RSS*_n_*H, RSS*_n_*R, *n* ≥ 2), is a common component found in mammalian cells, (Fukuto *et al*., 2018). It plays important physiological roles in cytoprotection, anti‐inflammatory activities and angiogenesis (Lau and Pluth, 2019) and is produced by various enzymes. Sulfide: quinone oxidoreductase (SQR) oxidizes H_2_S to hydrogen polysulfide (HS_n_H). Cystathionine beta‐lyase (CBS), cystathionine gamma‐lyase (CSE), 3‐mercaptopyruvate sulfurtransferase (3‐MST) and cysteinyl‐tRNA synthetase 2 (CARS2) produce sulfane sulfur from cysteine and its derivatives. Sulfane sulfur can be oxidized by persulfide dioxygenase (PDO; Hildebrandt and Grieshaber, 2008; Liu *et al*., 2014; Shen *et al*., 2015) or be reduced to sulfide by cellular thiols, thioredoxin and glutaredoxin (Hou *et al*., 2019).

Sulfane sulfur and its metabolizing enzymes have been studied in microorganisms as well, demonstrating that it is a key intermediate of H_2_S oxidation pathway (Luebke *et al*., 2014; Li *et al*., 2017b; Shimizu *et al*., 2017). Sulfane sulfur also modifies some gene regulators, a process now termed as protein S‐sulfhydration or persulfidation, to activate the expression of SQR and/or other H_2_S metabolizing enzymes. Four types of such gene regulators (CstR, BigR/SqrR, FisR and OxyR) have been identified (Giedroc, 2017; de Lira *et al*., 2018; Hou *et al*., 2019; Shimizu and Masuda, 2020). A recent research indicates that sulfane sulfur is involved in the regulation of pathogenicity in *Staphylococcus aureus* (Peng *et al*., 2017), suggesting that it has other physiological functions in bacteria.

Actinomycetes are remarkable producers of polyketide drugs, including erythromycin A, rifamycin, chromomycin and tetracyclines (Robertsen and Musiol‐Kroll, 2019). Advancements in microbial genomics indicate that earlier discoveries cite only a small fraction of polyketides in actinobacteria, representing approximately 10% of their biosynthetic capacity (Nett *et al*., 2009; Shen, 2015; Blin *et al*., 2019). Additionally, programmes related to isolation of natural products have significantly decreased since the end of the 21st century (Li and Vederas, 2009; Wright, 2017). One of the reasons responsible for the dearth of research on this subject is the fact that in actinobacteria, many biosynthetic gene clusters are regulated via unknown mechanisms. Their activation is on an ‘as‐needed’ basis; however, identifying the need is difficult (Bibb, 2013; Barka *et al*., 2016).

*Streptomyces coelicolor* M145 is a model strain for studying actinomycetes. It produces several polyketide compounds (Bentley *et al*., 2002; Hopwood, 2007), including blue‐pigmented actinorhodin (ACT; Bystrykh *et al*., 1996), red‐pigmented undecylprodigiosin and yellow‐pigmented coelimycin P2 (Liu *et al*., 2013; Chen *et al*., 2016). Among them, ACT has served as an outstanding example for genetic and biochemical investigations of polyketide metabolism, including the seminal work on polyketide biosynthetic gene clusters and the generation of hybrid polyketides (Strohl and Connors, 1992; Khosla *et al*., 1993). Although transcription regulation of the ACT biosynthetic gene cluster has been intensively studied, the intracellular signal that activates ACT production is still unclear (Liu *et al*., 2013; Mak and Nodwell, 2017).

In this study, we found that exogenous sulfane sulfur could function as a signal to activate ACT production in *S. coelicolor* M145. Endogenous sulfane sulfur was detected in this bacterium, and a natural gene circuit controlling its level was identified. Genetically editing the gene circuit changed the intracellular sulfane sulfur levels, ACT production and morphological development of *S. coelicolor* M145. The gene circuit contains elements that can sense and control intracellular sulfane sulfur level, to maintain it in a homeostatic state like a thermostat. This study unveiled new physiological roles of sulfane sulfur that may provide a new strategy for activating cryptic polyketide gene clusters in actinomycetes.

## Results

### Treating *S. coelicolor* with sulfane sulfur promoted its ACT production and spore formation

**Fig. 1:**
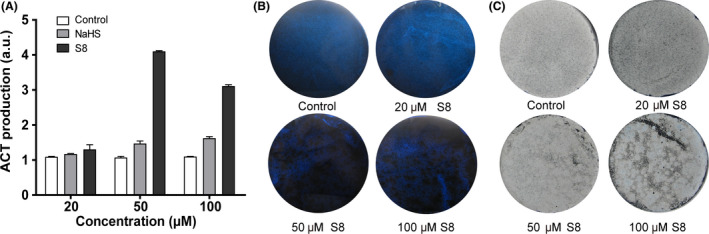
Using NaHS and S_8_ to treat *S. coelicolor* M145. Different concentrations of NaHS or S_8_ (20, 50 and 100 µM) were added to agar plates before inoculation of *S. coelicolor* M145 spores. Control was the plate without addition of the chemicals. After inoculation, the plates were incubated at 30 °C for 6 days and then subjected to analysis. A. Total ACT production of *S. coelicolor* M145. Data were from three repeats. B. S_8_ increased ACT production of *S. coelicolor* M145. Images were captured from the reverse side of the plates. C. S_8_ affected spore formation of *S. coelicolor* M145. Images were captured from the front side of the plates.

We used both sulfide (in the form of sodium hydrosulfide [NaHS]) and sulfane sulfur (in the form of S_8_) to treat *S. coelicolor* M145. These chemicals were mixed with the yeast‐beef‐peptone (YBP) agar and poured on a culture plate. Next, *S. coelicolor* M145 spores were spread on the surface of the plate, and the plate was incubated at 30 °C for 6 days. Addition of sulfide marginally increased ACT production, while addition of S_8_ significantly increased the production in the bacterial cells compared with that of untreated control cells (Fig. 1A and B). The highest increase was observed with the addition of 50 μM S_8_, about fourfold higher than the control. The addition of S_8_ also influenced morphological development of *S. coelicolor* M145, as evidenced by more spores being produced on the S_8_‐containing plate (Fig. 1C). These results demonstrated that sulfane sulfur can act as a stimulus to affect ACT production and spore formation of *S. coelicolor* M145.

### Endogenously produced sulfane sulfur also promoted ACT production and spore formation

**Fig. 2:**
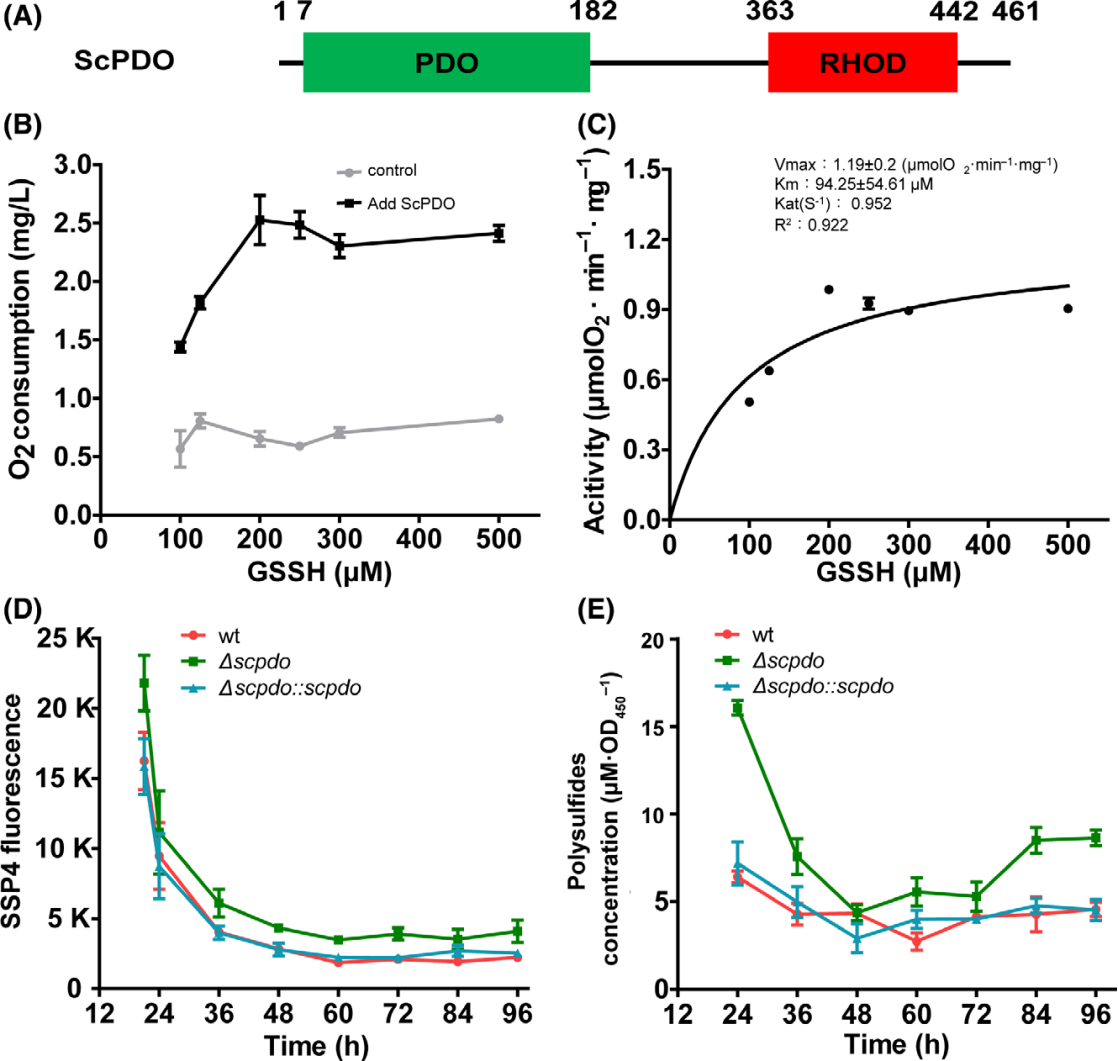
ScPDO had PDO activity, and its deletion increased intracellular sulfane sulfur. A. ScPDO had two hypothetical domains, PDO and RHOD. B and C. ScPDO displayed persulfide dioxygenase activity by oxidizing GSSH into sulfite. (B) Persulfide dioxygenase activity was confirmed by detecting O_2_ consumption. ScPDO (1 µM)_and 0–500 µM GSSH were mixed in 3 ml KPi buffer (100 mM, pH 7.4) at 25 °C, and O_2_ consumption was examined using an Orion RDO meter. Black line, reaction mixture with ScPDO; grey line, reaction mixture without ScPDO. (C) Catalysing kinetics of ScPDO was calculated using the Michaelis–Menten equation as reported previously (Liu *et al*., 2014). D and E. Detection of intracellular sulfane sulfur of wt, Δ*Scpdo and* Δ*Scpdo::Scpdo* strains with SSP4 and HPLC. High SSP4 fluorescence indicated high sulfane sulfur concentration in the cells. For B–D, data are expressed from three independent repeats and shown as mean ± SD.

Persulfide dioxygenase oxidizes glutathione persulfide (GSSH) and can lower cellular sulfane sulfur (Xia *et al*., 2017). The *S. coelicolor* M145 genome contains a gene coding for a hypothetical protein, SCO0618, containing an N‐terminal PDO‐like domain and a C‐terminal rhodanese domain (Fig. 2A). This indicates that SCO0618 might be a type III PDO. We compared it with three representative type III PDOs, *Zunongwangia profunda* SM‐A87 (ZpPDOIII, ADF52140.1), *Staphylococcus aureus* (SaPDOIII, WP_000465474.1) and *Bacillus cereus* ATCC 10876 (BcPdoIII, EEK49737.1) by using ClustalW (Fig. S1). To test whether this protein catalyses sulfane sulfur oxidation, we cloned its ORF into a pET15b vector and transformed the recombinant plasmid into *E. coli* BL21(DE3) for expression. The enzyme thus obtained was purified with an N‐terminal His‐tag using affinity chromatography and was confirmed via SDS‐PAGE (Fig. S2). Subsequent enzymatic assays indicated that it oxidized sulfane sulfur. When glutathione persulfide (GSSH) was used as a substrate, its *V*_max_ reached 1.19 ± 0.2 μmol O_2_ min^−1^ mg^−1^ and *K*_m_ value was 94.25 ± 54.61 μmol (Fig. 2B and C). Hereafter, we refer to this enzyme as ScPDO.

We deleted the *Scpdo* gene in *S. coelicolor* M145. *S. coelicolor* M145 wild type (wt) and the mutant Δ*Scpdo* did not display a significant difference in growth on agar plates. We subsequently detected its intracellular sulfane sulfur levels using a fluorescence‐based chemical probe SSP4 using a procedure described by Chen and colleagues (2013). The mutant Δ*Scpdo* accumulated a higher amount of sulfane sulfur, especially at the early exponential phase, than did wt (Fig. 2D). To confirm these results, we used an HPLC‐based method to quantify the intracellular sulfane sulfur in both Δ*Scpdo* and wt (Ran *et al*., 2019). The mutant accumulated 16.06 ± 0.41 μM·OD_450_^−1^ intracellular sulfane sulfur at 24 h, while wt accumulated 6.41 ± 0.34 μM·OD_450_^−1^ (Fig. 2E). We also constructed a complemented strain (Δ*Scpdo:: Scpdo*) by introducing an *Scpdo*‐expression plasmid into Δ*Scpdo*, in which the *Scpdo* expression was controlled by a constitutive promoter, kasOp* (Bai *et al*., 2015). This strain produced similar amounts of intracellular sulfane sulfur as wt. These results indicated that ScPDO was indeed responsible for sulfane sulfur oxidation, preventing the accumulation of sulfane sulfur inside the cells. Notably, in all three strains, sulfane sulfur accumulation showed a similar trend; the level of sulfane sulfur was the highest at the exponential phase, then started to decrease and finally stabilized at the stationary phase (Fig. 2D and E).

**Fig. 3:**
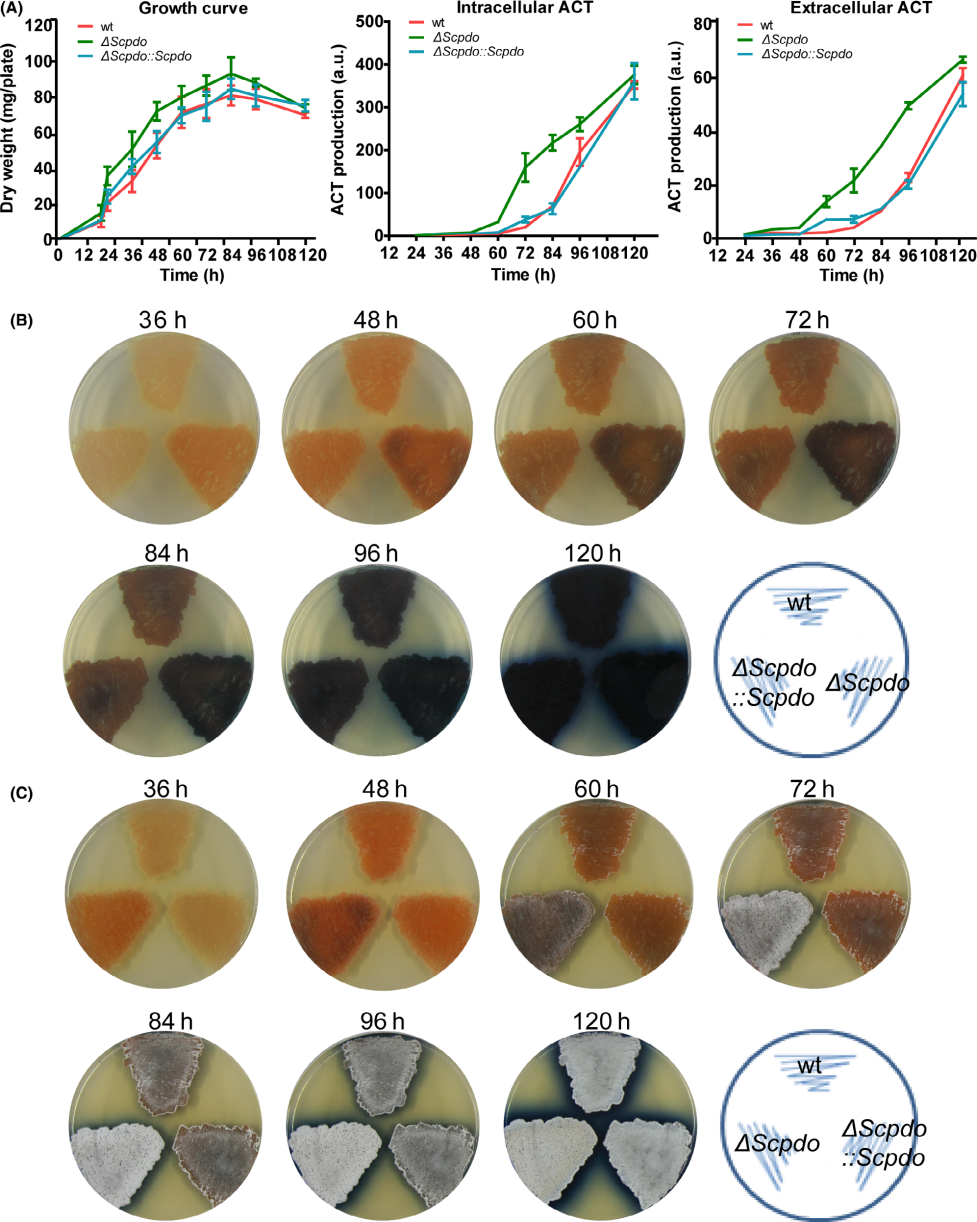
Deletion of ScPDO increased ACT production and spore formation of *S. coelicolor* M145. A. Growth and ACT production of the strains in agar plates. Both intracellular and extracellular ACT production were quantified. Dry weight of mycelia (mg plate^−1^) was quantified and used to represent the growth curve of *S. coelicolor* M145. The error bars are the standard deviation of the data obtained from three replicates. For comparison, equal amounts of spores of wt, Δ*Scpdo* and Δ*Scpdo:: Scpdo* strains were inoculated on the YBP agar medium and cultured at 30 °C for 5 days. Intracellular ACT was quantified using only the mycelia, and extracellular ACT was quantified using only the medium, as reported previously (Kieser *et al*., 2000). Data are expressed from three independent repeats. B and C. Δ*Scpdo* strain showed higher ACT production and spore formation than wt and Δ*Scpdo:: Scpdo* strains; images were captured from reverse and front sides of the plates respectively.

We observed that Δ*Scpdo* produced more ACT than wt, especially from 60 to 96 h (Fig. 3A and B). Quantitative analysis indicated that the ACT production by Δ*Scpdo* was about two‐ to sevenfold higher than that of wt depending on the cultivation time. In addition, Δ*Scpdo* generated spores significantly earlier than wt and Δ*Scpdo:: Scpdo* (Fig. 3C). However, the Δ*Scpdo:: Scpdo* strain exhibited a similar trend of ACT production as wt.

### Identification of a transcription factor governing intracellular sulfane sulfur levels

**Fig. 4:**
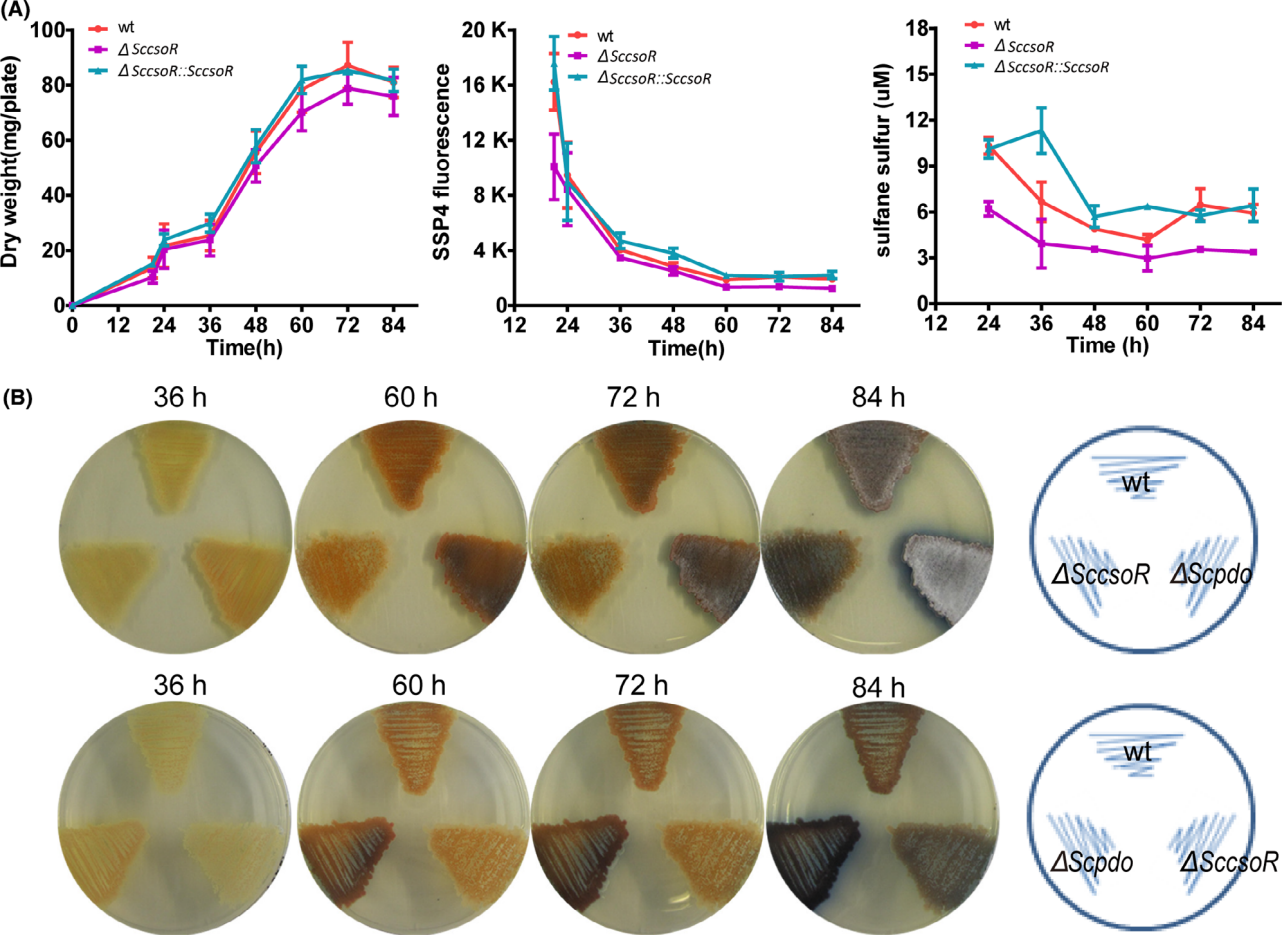
Deletion of ScCsoR decreased intracellular sulfane sulfur and decreased both ACT production and spore formation. A. Growth and sulfane sulfur production of *S. coelicolor* M145. The dry weight of mycelia (mg/plate) was quantified and used to represent the growth curve of *S. coelicolor* M145. For comparison, equal amount spores of wt, Δ*SccsoR* and Δ*SccsoR:: SccsoR* strains were inoculated on YBP agar medium and cultured at 30 °C for 84 h. Intracellular sulfane sulfur of *S. coelicolor* M145 strains was detected using both SSP4 and HPLC. Low SSP4 fluorescence indicated low sulfane sulfur concentration in the cells. Data were from three independent repeats and shown as average ± SD. B. The Δ*SccsoR* strain showed lower ACT production and spore formation than wt and Δ*Scpdo* strains. Photographs were taken from reverse and front sides of the plates.

Genome background analysis indicated that there is a hypothetical transcription factor gene (*sco0620*) adjacent to *Scpdo*, which encodes a CsoR‐like_DUF156 protein. Hereafter, we refer to this protein and its encoding gene as ScCsoR and *SccsoR* respectively. To test whether ScCsoR is involved in the regulation of intracellular sulfane sulfur levels, we constructed a *SccsoR* deletion strain Δ*SccsoR* and a complemented strain Δ*SccsoR:: SccsoR*. SSP4 test showed that Δ*SccsoR* accumulated slightly less sulfane sulfur than wt, whereas Δ*SccsoR:: SccsoR* produced slightly more sulfane sulfur than wt. The production differences were more apparent with the HPLC test (Fig. 4A). Moreover, Δ*SccsoR* produced less ACT than wt and its sporulation was also significantly delayed (Fig. 4B).

**Fig. 5:**
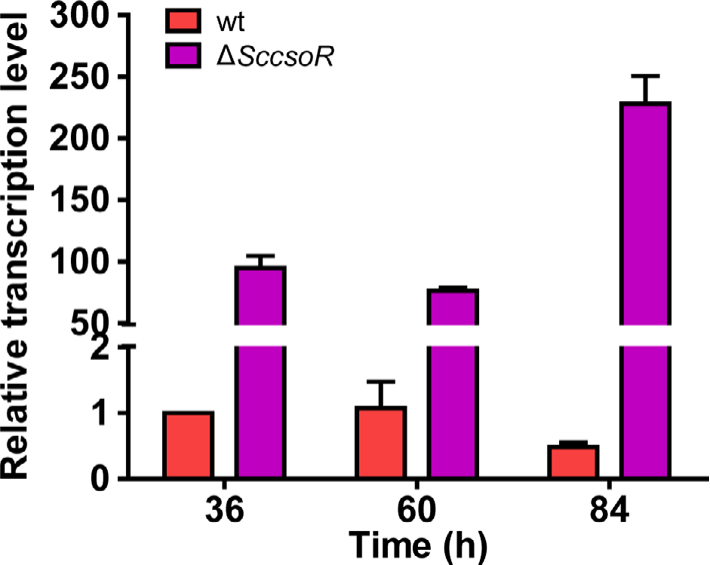
The *Scpdo* gene showed higher transcription level in Δ*SccsoR* strain than in wt strain. RT‐qPCR analysis was performed to compare the transcriptional level of *Scpdo* in wt and Δ*SccsoR* strains. RNA was harvested at 36, 60 and 84 h in YBP agar medium. *hrdB* transcription was used as the internal control for normalization. Three independent measurements were carried out; error bars indicate standard deviation.

To test whether ScCsoR functions via regulating the expression of *Scpdo*, we analysed the *Scpdo* transcription levels in both Δ*SccsoR* and wt using RT‐qPCR. The level of *Scpdo* mRNA in Δ*SccsoR* was much higher than that in wt, with Δ*SccsoR* exhibiting 100‐ to 480‐fold higher levels than wt depending on the growth time (Fig. 5). These results suggested that ScCsoR negatively controlled the expression of *Scpdo*.

### ScCsoR is a transcription repressor that binds to the upstream sequence of Scpdo promoter

**Fig. 6:**
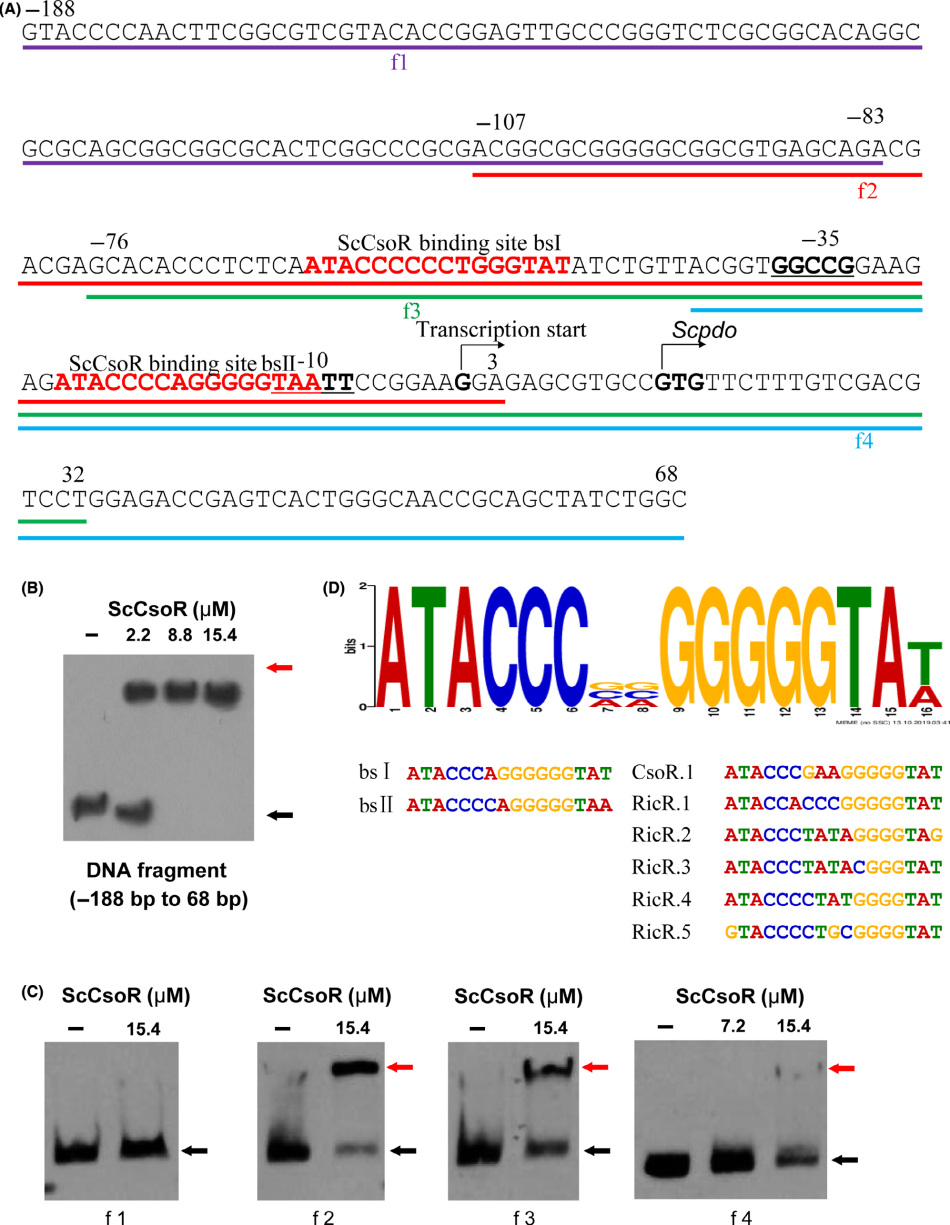
Characterization of the *Scpdo* promoter. A. DNA sequence of *Scpdo* promoter. The transcription start site of *Scpdo* was identified using 5’‐RACE. GTG is the start codon. The DNA fragments used for EMSA analysis were denoted as f1 to f4. B. EMSA analysis of the binding affinity of ScCsoR (in reduced form) to *Scpdo* promoter. DNA probe (1 nM) was incubated with different amounts of ScCsoR (0, 2.2, 8.8, 15.4 µM). Black arrow indicates the free DNA probe, and red arrow indicates ScCsoR‐DNA complex. C. EMSA analysis of the ScCsoR (in reduced form) binding affinity to different parts of *Scpdo* promoter. The DNA probe used in (B) was divided into four DNA fragments (f1 to f4), and other conditions were the same as in (B). Only f2 and f3 fragments exhibited obvious band shifts after incubation with ScCsoR, suggesting that ScCsoR was bound to the promoter region of *Scpdo* gene. D. Multiple EM for Motif Elicitation analysis of *Scpdo* promoter. The binding sequences of CsoR from *Geobacillus thermodenitrificans* and RicR from *Mycobacterium tuberculosis*, and sequences of f2, f3 fragments were used as inputs for MEME analysis. A 16‐nt consensus sequence with reverse palindrome was identified (ATACCn6GGTAT).

**Fig. 7:**
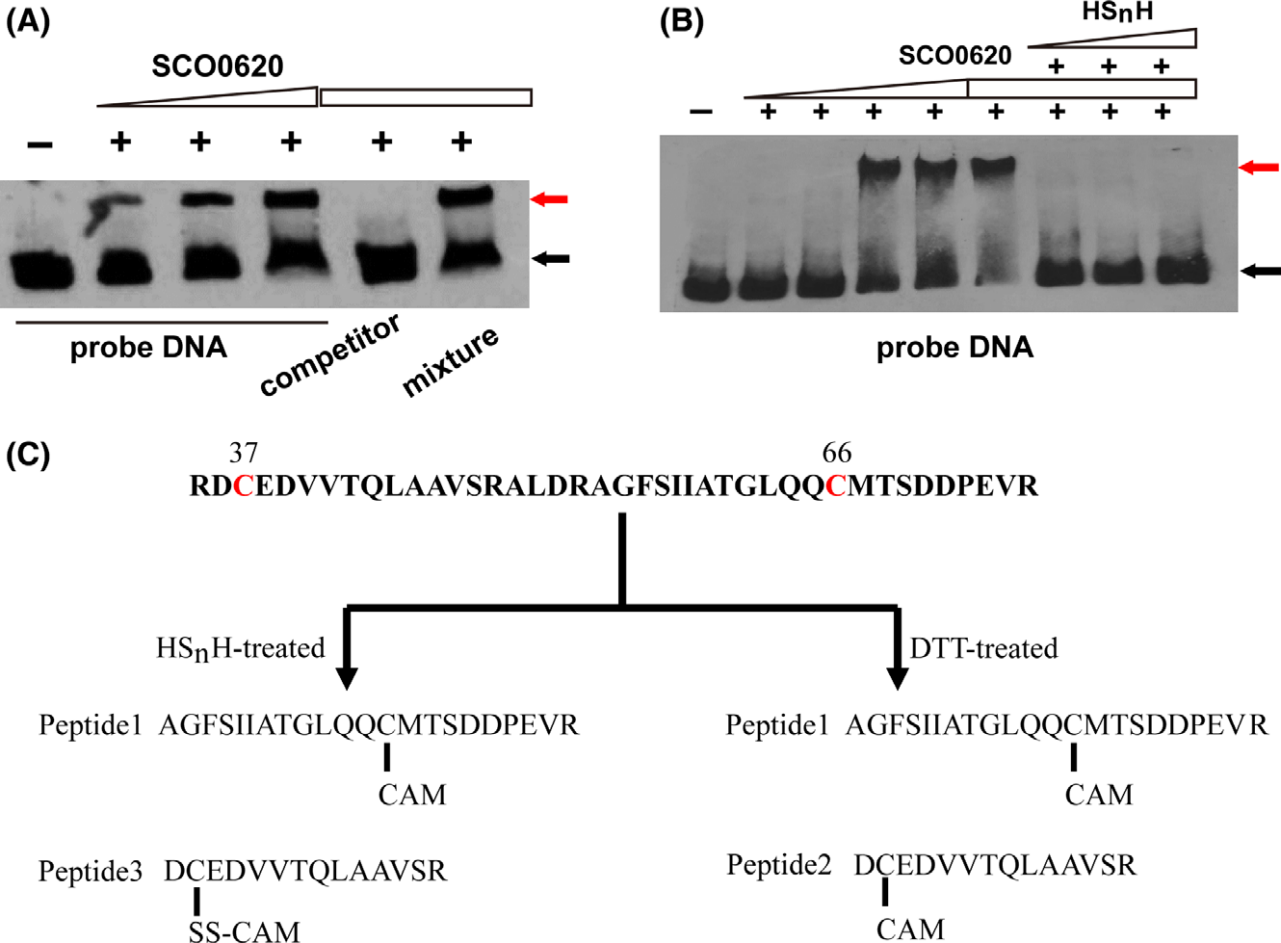
Hydrogen polysulfide (HS_n_H) affected the binding affinity of ScCsoR to *Scpdo* promoter. A. ScCsoR exhibited binding to the probe DNA containing bsΙ and bsΠ, even in the presence of competitor DNA. DNA probe (1 nM) was incubated with different amounts of ScCsoR (0, 2.2, 8.8, 15.4 µM). Black arrow indicates the free DNA probe, and red arrow indicates the shifted DNA probe. 50‐fold excess of unlabelled competitor DNA and its mixture with probe DNA were also tested. B. HS_n_H detached ScCsoR from the probe DNA. DNA probe (1 nM) was incubated with different concentrations of ScCsoR (0, 0.5, 1.0, 2.0, 4.0, 8.0, 8.0, 8.0, 8.0 µM). Different amounts of HS_n_H (0, 1, 2, 3 mM) were added to the reaction system. C. LC‐MS/MS analysis of HS_n_H‐ and DTT‐treated ScCsoR. Details of the MS data are shown in Figs S3–S5.

To investigate the mechanism by which ScCsoR controls the expression of *Scpdo*, we expressed *SccsoR* gene in *E. coli* BL21(DE3) and obtained the purified ScCsoR protein. A 5'‐rapid amplification of cDNA ends (RACE) experiment indicated that *Scpdo* transcription started at −12 bp relative to the start codon GTG. Subsequently, a 256‐bp DNA fragment containing the promoter and partial ORF sequence of *Scpdo* was synthesized (Fig. 6A). Purified ScCsoR showed significant affinity to this DNA fragment, as judged by an EMSA experiment (Fig. 6B). To determine the exact binding site of ScCsoR, we synthesized four short DNA fragments (f1–f4), with each fragment containing a segment of the 256‐bp DNA (Fig. 6A). EMSA experiments indicated that ScCsoR preferred to bind f2 and f3 fragments (Fig. 6C). We used the Multiple EM for Motif Elicitation (MEME; http://meme‐suite.org/) toolbox to analyse the f2 and f3 fragments and identified a 16‐bp segment containing a pair of palindromic sequences (ATACCn6GGTAT). The 16‐bp segment showed a high similarity to the binding sites of *Geobacillus thermodenitrificans* CsoR (Coyne and Giedroc, 2013; Chang *et al*., 2014) and *Mycobacterium tuberculosis* RicR (Festa *et al*., 2011; Shi *et al*., 2014; CsoR‐like Transcription factor; Fig. 6D), suggesting that it is the binding site of ScCsoR. There are two ScCsoR binding sites in the *Scpdo* promoter. We designated them as bsΙ and bsΠ, located at ‐ 48 and ‐ 9 sites, respectively, from the transcription start site. For further confirmation, we examined the binding affinity of ScCsoR to a 79‐bp DNA fragment containing bsI, bsΠ, as well as to an intergenic sequence located between them. A competitor DNA that did not contain these sequences was used as the control. ScCsoR bound to the 79‐bp DNA but not to the competitor DNA (Fig. 7A); in addition, when the 79‐bp DNA fragment was mixed with 50‐fold (mole ratio) of the competitor DNA, ScCsoR still bound specifically to this fragment.

### ScCsoR detaches from Scpdo promoter in the presence of sulfane sulfur

We performed an EMSA experiment in the presence of hydrogen polysulfide (HS_n_H). The binding affinity of ScCsoR was significantly decreased by HS_n_H (Fig. 7B). Thus, these results indicated that sulfane sulfur reacted with ScCsoR, leading to its detachment from the DNA binding site.

Previous studies have demonstrated that transcription factors CstR, BigR/SqrR, FisR and OxyR all use their cysteine residues to react with sulfane sulfur (Giedroc, 2017; Li *et al*., 2017b; Hou *et al*., 2019). ScCsoR contains two cysteine residues, Cys37 and Cys66. To explore which one of these is required to sense sulfane sulfur, we treated the purified ScCsoR with HS_n_H and analysed the treated protein with LC‐MS/MS. As a reference, DTT‐treated ScCsoR was also analysed following the same protocol. Two peptides were found in the DTT‐treated sample: peptide 1 contained Cys66, and peptide 2 contained Cys37 (Fig. 7C; Figs S3 and S4). Their thiol groups were directly blocked by acetamide (CAM), indicating that these two peptides were not modified. Peptide 1 was also found in the HS_n_H‐treated sample, and a third peptide, peptide 3, which contained Cys37‐SS‐CAM modification, was found in the HS_n_H‐treated sample but not in the DTT‐treated sample (Fig. S5), indicating that Cys37‐SH could be modified to Cys_37_‐SSH when ScCsoR was treated with sulfane sulfur.

We then constructed three ScCsoR mutants: ScCsoR‐C37S, ScCsoR‐C66S and ScCsoR‐C37S‐C66S, in which the cysteine residue was mutated to serine. EMSA results indicated that these three mutants could still bind to the probe DNA; however, HS_n_H could not detach them from the probe DNA (Fig. S6). These results indicated that both Cys37 and Cys66 were involved in the sulfane sulfur sensing process of ScCsoR.

### ScCsoR is a regulator of a sulfane sulfur oxidation gene circuit

**Fig. 8:**
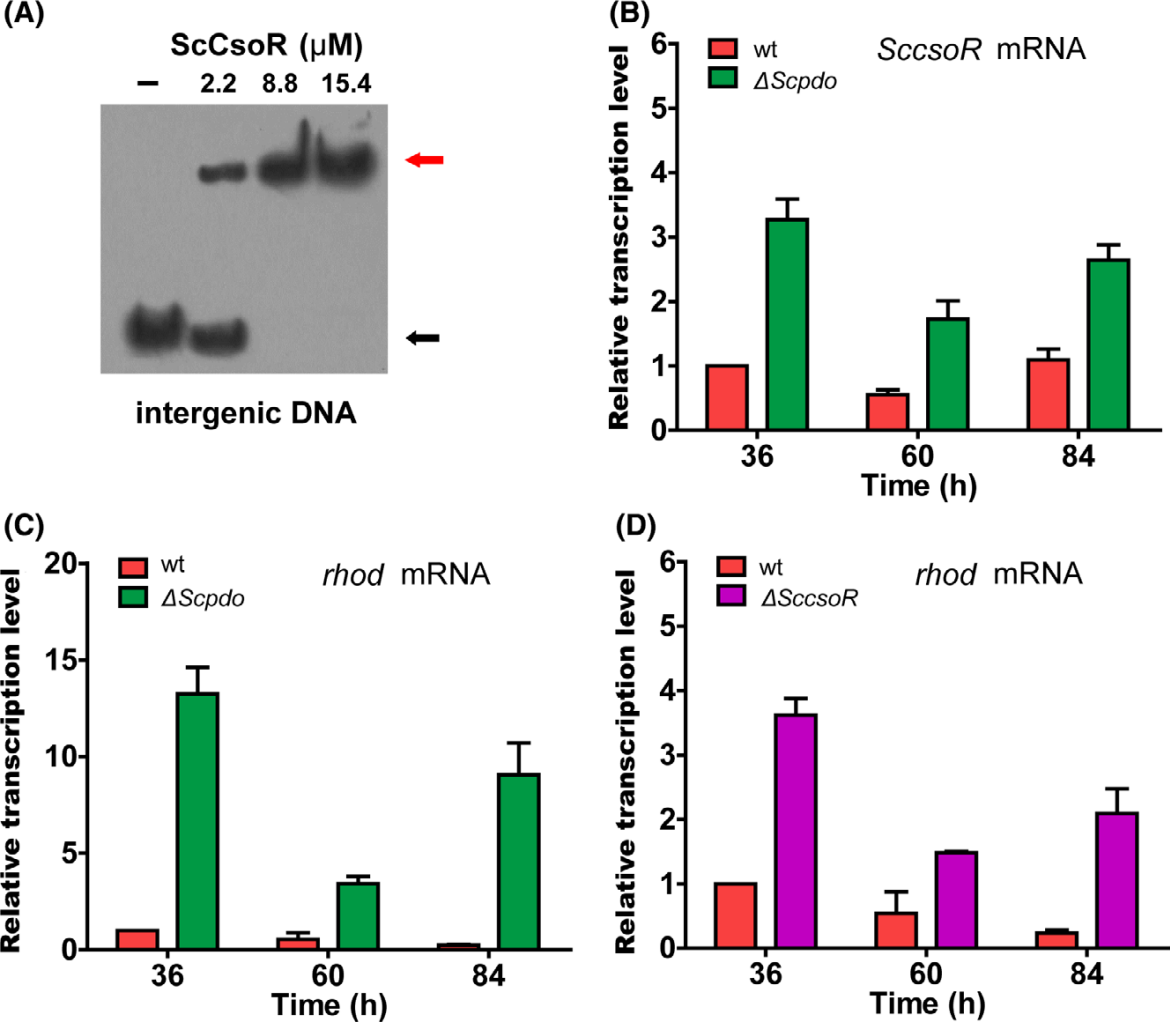
ScCsoR controlled transcription of *SccsoR* and *Scrhod*. A. EMSA analysis of ScCsoR binding affinity to the intergenic DNA located between *SccsoR* and *Scrhod*. The intergenic region of *SccsoR* and *Scrhod* (261bp) was used as a probe. DNA (1 nM) probe was incubated with different concentrations of ScCsoR (0, 2.2, 8.8, 15.4 µM). Black arrow indicates the free DNA probe, and red arrow indicates the shifted DNA probe. B. RT‐qPCR analysis of *SccsoR* mRNA in Δ*Scpdo* strain. C. RT‐qPCR analysis of *Scrhod* mRNA in Δ*Scpdo* strain. D. RT‐qPCR analysis of *Scrhod* mRNA in Δ*SccsoR* strain. For B‐D, data are expressed from three independent repeats. For B, C, D, RNA was harvested at 36, 60 and 84 h in YBP agar medium. The *hrdB* transcription was used as the internal control for normalization. Three independent measurements were carried out; error bars indicate standard deviation

We found that the 16‐bp binding site is also present in the intergenic region of *SccsoR* and *sco0621* promoters (Fig. S7); the latter encode a hypothetic protein containing a Rhod homology domain (Motl *et al*., 2017). Hereafter, we refer to this gene as *Scrhod*. EMSA experiments confirmed that ScCsoR could bind to the intergenic region (Fig. 8A). Moreover, RT‐qPCR experiments indicated that the expression levels of both *SccsoR* and *Scrhod* were increased in the sulfane sulfur over‐accumulation strain (Δ*Scpdo*; Fig. 8B and C). In addition, *Scrhod* expression was also increased because of the deletion of *SccsoR* (Fig. 8D). These results indicated that both the transcription of *SccsoR* and *Scrhod* was regulated by ScCsoR.

The gene adjacent to *Scpdo is sco0619*, which encodes a possible membrane protein homologous to the sulfite exporter, TauE (Weinitschke *et al*., 2007). Hereafter, we refer to this gene as *SctauE*. RT‐PCR experiments indicated that *Scpdo and SctauE* were co‐transcribed (Fig. S8A). These analyses suggested that ScCsoR not only controlled the oxidation of cellular sulfane sulfur, but also controlled secretion of the oxidized product, sulfite.

RT‐PCR revealed that *Scrhod* was not co‐transcribed with *sco0622* (Fig. S8A). EMSA experiments demonstrated that ScCsoR could not bind to the promoter of *sco0622* (Fig. S8B). RT‐qPCR further demonstrated that *sco0622* and *sco0623* were co‐transcribed and their expression was not affected by the *SccsoR* deletion (Fig. S8C), indicating that *sco0622* and *sco0623* were not controlled by ScCsoR, thereby clarifying the boundary of the ScCsoR controlled gene circuit.

### Distribution of sulfane sulfur oxidation gene circuit in other Streptomyces species

**Fig. 9:**
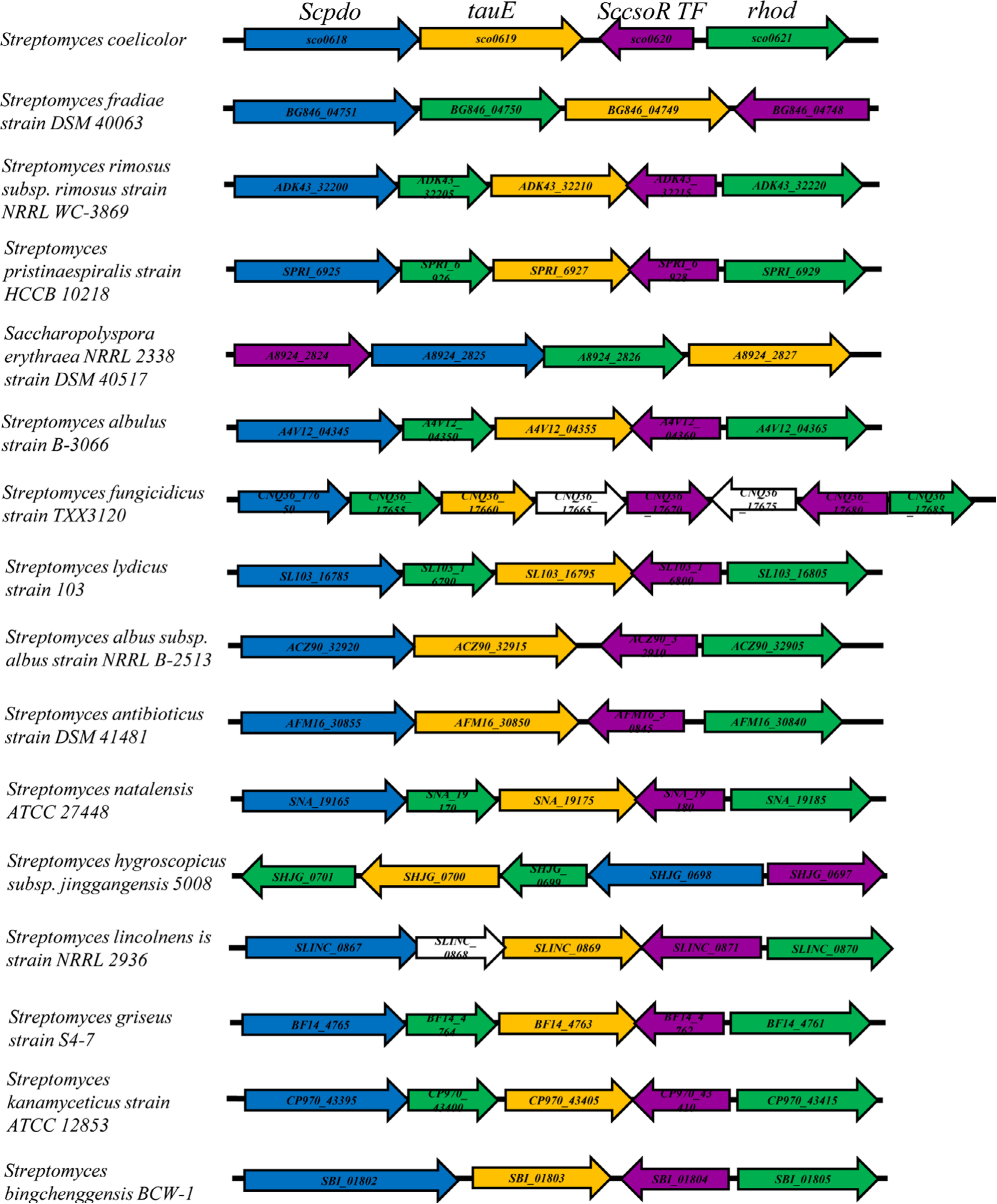
Hypothetical sulfane sulfur oxidation gene circuits found in 16 species of *Streptomyces*. Details of the strains are provided in Table S1.

To investigate whether a similar gene circuit is present in other species of *Streptomyces*, we analysed all the sequenced *Streptomyces* genomes deposited at the website https://patricbrc.org/, which contained 1977 genomes as ascertained on Nov 26, 2019. ScPDO homologous proteins were found in 1248 genomes. Among them, 773 genomes also contained the genes coding for ScCsoR homologous proteins (CsoR‐like TF) close to their *pdo* genes (within 3‐ORF distance). We further analysed the genomes of 16 strains that were widely studied due to the production of important antibiotics and various polyketide drugs (Table S1), including the neomycin producer *S. fradiae*, validamycin producer *S. hygroscopicus*, lincomycin producer *S. lincolnensis* and natamycin producer *S. lydicus*. They were all observed to harbour a similar gene circuit containing PDO, TauE, RHOD and CsoR‐like TF as that of ScPDO (Fig. 9). These results suggested that the phenomenon of cellular sulfane sulfur affecting polyketide biosynthesis might be widely present in the *Streptomyces* species.

## Discussion

**Fig. 10:**
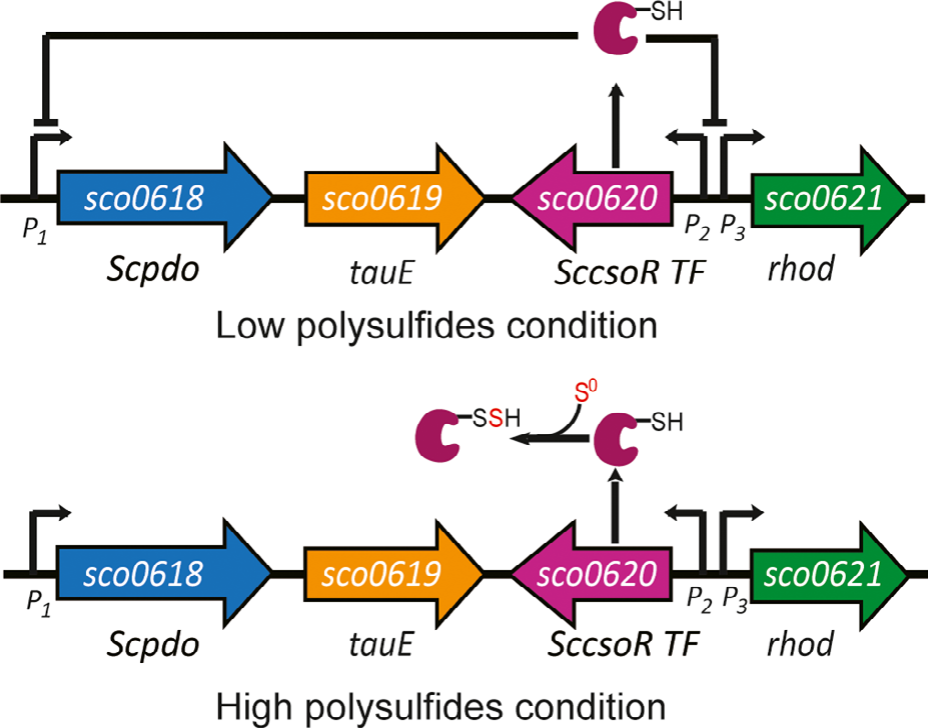
Functioning mechanism of the ScCsoR‐pivoted gene circuit. Reduced ScCsoR (Cys37‐SH) binds to the promoters of *Scpdo* (P1), *SccsoR* (P2) and *rhod* (P3) and inhibits their expression. When the intracellular sulfane sulfur level reaches its threshold, ScCsoR is sulfhydrated (Cys37‐SSH) by sulfane sulfur and dissociates from the promoters, allowing the expression of these genes until the intracellular sulfane sulfur level drops below the threshold.

We identified a gene circuit comprising four genes that specifically oxidizes sulfane sulfur in *S. coelicolor* M145. On the basis of our results, we have proposed a functioning mechanism for this gene circuit (Fig. 10). ScCsoR is a regulator that represses the expression of all the four genes including it. It exhibits a 46% homology with *Staphylococcus aureus* CstR is (Fig. S9) and contains two conserved cysteine residues. However, the sensing mechanism of ScCsoR is different from that of CstR. CstR forms intra tri‐ and tetra‐sulfide bonds between its two cysteine residues when reacting with sulfane sulfur (Luebke *et al*., 2014). However, in the case of ScCsoR, no such bonds are formed and only its Cys37 is persulfidated (Cys_37_‐SSH), leading to its detachment from the promoters. Interestingly, although Cys66 is not persulfidated by sulfane sulfur, it is also involved in the detachment process, probably via stabilizing Cys_37_‐SSH, as a role of Cys208 in OxyR (Hou *et al*., 2019). ScPDO oxidizes sulfane sulfur to sulfite; the latter is exported, probably by TauE. The action of ScPDO and TauE lowers cellular sulfane sulfur. Since the expression of ScCsoR is self‐regulated and is expressed in the presence of high cellular sulfane sulfur, when the sulfane sulfur levels become low, the newly produced ScCsoR do not become persulfidated and quickly bind to the promoters to repress the expression of these genes. This provides a feedback mechanism, representing a new type of regulation, specifically for maintaining the sulfane sulfur homeostasis in bacteria.

This gene circuit is different from previously characterized gene circuits responsible for H_2_S oxidation in bacteria (Luebke *et al*., 2014; Li *et al*., 2017b; Shimizu *et al*., 2017). The H_2_S‐oxidizing circuits possess both SQR and PDO. SQR oxidizes H_2_S to sulfane sulfur, which is sensed by the gene regulator to elevate the expression of *sqr* and *pdo*. Bioinformatic analyses indicate that both H_2_S oxidation and sulfane sulfur oxidation gene circuits are widely present in heterotrophic bacteria (Xia *et al*., 2017; de Lira *et al*., 2018). Our study indicates that the regulation mechanisms of H_2_S oxidation and sulfane sulfur oxidation are different. We observed that all the TFs in the H_2_S‐oxidizing gene circuits are constitutively expressed at low levels and that these circuits are very sensitive even to low levels of H_2_S (< 10 μM; Xia *et al*., 2017; Li *et al*., 2017a), suggesting that bacteria containing H_2_S‐oxidizing gene circuits cannot tolerate the presence of H_2_S. However, the expression of TF of the sulfane sulfur oxidation gene circuit is dynamic, which can confer wide amplitude of expression on the genes that it controls – from being completely silenced to be fully turned‐on. This makes the strain tolerant to certain levels of intracellular sulfane sulfur – the gene circuit is turned on by a threshold level.

We found that sulfane sulfur affects ACT production as well as spore formation in *S. coelicolor* M145. Further investigations are needed to illustrate its exact functioning mechanism. For ACT production, hundreds of genes are involved in this process (Xu *et al*., 2017; Xu *et al*., 2019). Among them, ActII‐ORF4 has been identified as a determining regulator and its expression is regulated by at least eight regulatory proteins (Liu *et al*., 2013). Our preliminary study indicated that sulfane sulfur cannot directly react with ActII‐ORF4, suggesting that sulfane sulfur possibly reacts with a regulatory protein that controls ActII‐ORF4 (data not shown). For the generation of spores, the regulation is more complex than that of ACT production. Spore formation involves more than a dozen regulatory proteins that compose a multiple‐layered regulatory network (Bush *et al*., 2015). Investigating which of these proteins are affected by sulfane sulfur requires systematic studies, including transcriptomics and/or proteomics approaches. Further, sulfane sulfur levels can also be controlled multiple mechanisms. We observed that sulfane sulfur levels in the Δ*Scpdo* strain varied with the time of cultivation, indicating that the ScCsoR‐pivoted gene cluster is not the only system that acts as an effector. The intracellular redox potential controlled by the ratio of reduced mycothiol/oxidized mycothiol could be another effector (Newton *et al*., 2008). Thus, the sulfane sulfur metabolism, ACT production and sporulation might be interrelated in *S. coelicolor* M145.

Our finding is significant with respect to the study of polyketides as well as antibiotics. There are many undeveloped polyketide biosynthetic gene clusters in acetinomycetes of which the regulatory mechanisms are yet unknown. Using genetic engineering methods (most commonly by altering their promoters) to activate these gene clusters usually involves laborious work and are not always effective. Sulfane sulfur may represent a new stimulus that can activate certain polyketide biosynthetic gene clusters. Using exogenous sulfane sulfur to treat acetinomycetes or altering their intracellular sulfane sulfur might help in the discovery of new polyketide drugs from such bacteria.

## Experimental procedures

### Strains and growth conditions

All the strains and plasmids used in this study are listed in Table S2. *S. coelicolor* M145 and its derivatives were cultured at 30 °C. Mannitol soya flour (MS) agar medium was used for sporulation and conjugation. YBP (yeast‐beef‐peptone) solid medium was used for determining ACT production, phenotypic observation, growth analysis and RNA extraction. YBP liquid medium was used for quantifying intracellular sulfane sulfur. Antibiotics were added to the media when required. *E. coli* DH5α was used for plasmid construction, and the *E. coli* BL21 (DE3) strain was used for protein expression. *E. coli* ET12567 (pUZ8002) was used to transfer nonmethylated DNA into *S. coelicolor* M145.

### Construction of *S. coelicolor* Δ*Scpdo* and Δ*SccsoR*

All the primers used in this study are listed in Table S3.The mutant, Δ*Scpdo* of *S. coelicolor,* was constructed using the sgRNA‐guided CRISPR‐Cas9 genome editing system (Huang *et al*., 2015). Upstream (1008 bp) and downstream (1131 bp) regions of *Scpdo* ORF were amplified from *S. coelicolor* M145 genomic DNA. A 20‐nt NGG mode sequence (5′‐ACCGGCGGTTCGCTGCTGAT‐3′) was obtained from CRISPy‐web (http://crispy.secondarymetabolites.org/). A special single guide RNA (sgRNA; containing a 20‐nt sequence) was amplified from pKCcas9dO. The linear pKCcas9dO vector was generated by SpeI/HindIII digestion. The upstream and downstream DNA fragments, sgRNA and the linear pKCcas9dO vector were finally assembled to generate the plasmid pKCcas9dO‐sco0618 using the ClonExpress™ II One Step Cloning Kit (TaKaRa). This plasmid was introduced into *S. coelicolor* M145 by conjugal transfer between *E. coli* ET12567 (pUZ8002) and M145. The exconjugants were selected and examined following a reported protocol (Li *et al*., 2017a). After incubation at 37 °C for several rounds of selection, the pKCcas9dO‐*Scpdo* plasmid was extracted from the mutant and the final construct, Δ*Scpdo*, was obtained. The strain Δ*SccsoR* was subsequently constructed using a homologous recombination method (Lu *et al*., 2018).

### Construction of the complementary strains

To construct a complementary strain of Δ*Scpdo*, namely, Δ*Scpdo*:: *Scpdo*, a DNA fragment containing a kasOp* promoter (Bai *et al*., 2015) and *Scpdo* ORF was obtained using PCR. This DNA fragment was inserted into the integrative vector pMS82 to make a new plasmid pCom0618 (Table S2), which was then transformed into the Δ*Scpdo* strain using a conjugation method. The Δ*SccsoR* complementary strain, Δ*SccsoR*:: *SccsoR*, was constructed following the same protocol, except that a DNA fragment containing *SccsoR* ORF and its native promoter (a 507 bp fragment upstream of its ORF) was used. To construct control strains, empty pMS82 vectors were also transformed into Δ*Scpdo* and Δ*SccsoR* strains.

### Examination of the ACT production

For testing the effects of H_2_S and sulfane sulfur, *S. coelicolor* M145 spores (2 × 10^6^) were inoculated on YBP agar plates containing different concentrations of NaHS or S_8_, and the plates were incubated at 30 °C for 6 days. For quantitative analysis of the ACT production, a previously reported method was used (Kieser *et al*., 2000). Briefly, total ACT was quantified by scraping off all the medium containing mycelia from the plate and mixing it with 1 M KOH. The reaction was conducted overnight at 25 °C, and later, 1 ml aliquot was taken out for quantification. Intracellular ACT was quantified using only the mycelia, and extracellular ACT was quantified using only the medium. ACT in the sample was quantified by measuring absorbance at 640 nm.

### Analysis of intracellular sulfane sulfur

To perform the SSP4 test, *S. coelicolor* M145 and its derivatives (10 ml) were cultured in liquid YBP medium. The mycelia were harvested at different time points, washed with and finally suspended in HEPES buffer (50 mM, pH 7.4). Cells were broken down using a high‐pressure crusher SPCH‐18 (STANSTED). SSP4 (10 μM) was added to each supernatant, and the mixture was incubated at 37 °C for 15 min in the dark with gently shaking (125 rpm). Fluorescence intensity was measured using the SynergyH1 microplate reader. The excitation and emission wavelengths were set at 482 and 515 nm respectively. The final data were converted to fluorescence intensity per 0.5 OD_450_ of cells.

For HPLC analysis, a method describe previously was used (Ran *et al*., 2019). Briefly, cells at 1 OD_450_ were collected and washed with HEPES buffer (50 mM, pH 7.4) and then resuspended in 220 μl of 50 mM Tris‐HCl buffer (pH 9.5) containing 1% (v/v) Triton X‐100, 50 μM DTPA, and 1 mM sulfite. The samples were incubated at 95 °C for 10 min to convert sulfane sulfur to thiosulfate. After centrifugation, 50 μl supernatant was reacted with mBBr (5 μl) in the dark for 30 min, and 100 μl of acetic acid and acetonitrile mixture (v/v, 1:9) was added to stop the reaction. The finally obtained sample was analysed using HPLC equipped with a fluorescence detector.

### Protein mutation, expression and purification

The ORFs of *Scpdo and SccsoR* were amplified from *S. coelicolor* M145 genomic DNA. ScCsoR mutants were constructed using the revised QuikChange™ method (Xia *et al*., 2015). The ORFs were ligated into pET15b plasmid (Table S2) for expression. *E. coli* BL21(DE3) cells containing pET15b derived plasmids were cultured in LB medium at 37 °C until OD_600nm_ reached about 0.5, and then 0.5 mM isopropyl β‐D‐1‐thiogalactopyranoside (IPTG) was added. The temperature was changed to 30 °C, and the cultivation was continued overnight. Cells were harvested by centrifugation and disrupted using a pressure cell homogenizer (SPCH‐18) at 4 °C in buffer I (50 mM NaH_2_PO_4_, 250 mM NaCl, 20 mM imidazole, pH 8.0). The His‐tagged protein was purified using Ni‐NTA‐Sefinose column (Sangon) following the manufacturer’s instructions. The purity of the protein was examined using SDS‐PAGE, and its concentration was determined using a bicinchoninic acid assay.

### Assay of ScPDO activity

The persulfide dioxygenase activity of ScPDO was determined using a previously reported method (Xin *et al*., 2016). The reaction system contained 1 μM ScPDO and different concentrations of GSSH (0–0.5 mM) in 3 ml KPi buffer (100 mM, pH 7.4), and the reaction was conducted at 25 °C. O_2_ consumption was examined using an Orion RDO meter (Thermo Scientific). The kinetic parameters were calculated using the Michaelis–Menten equation as reported previously (Liu *et al*., 2014). GSSH was prepared as described previously (Xin *et al*., 2016).

### RT‐PCR, RT‐qPCR and 5'‐RACE analysis

RNA from *S. coelicolor* was prepared by following a previously described protocol (Lu *et al*., 2018). Mycelia were harvested at different time points (36, 60 and 84 h) and were ground into powder using liquid nitrogen. Total RNA was treated with TRIzol, and DNase Ι was used to obtain chromosomal DNA. RT‐PCR was carried out using a reverse transcriptase kit (Invitrogen). RT‐qPCR was performed using SYBR Premix Ex Taq (Takara). *hrdB* gene encoding a major sigma factor was used as control to normalize the relative quantities of cDNA. Roche LightCycler 480 thermal cycler was used to determine the melting curve and specificity of the PCR products. Three independent replicates were performed in parallel. 5'‐RACE was performed employing the FirstChoice RLM‐RACE kit (Thermo) following the manufacturer’s instructions.

### Electrophoretic mobility shift assay (EMSA)

DNA probes were labelled with 5ˊ‐Biotin, and 1 nM each of the labelled probes was mixed with differing amounts of purified ScCsoR protein in a binding buffer (20 mM Tris‐HCI, 2 mM EDTA, 20 mM KCI, 0.5 mM dithiothreitol (DTT) or 1 mM–3 mM HS_n_H, 4% (w/v) Ficoll‐400, pH 8.0). HS_n_H was prepared by mixing H_2_S with S_8_ as reported previously (Xin *et al*., 2016). The reaction was conducted at 25 °C for 15 min, followed by separation on an 8% (w/v) non‐denaturing polyacrylamide gel in an ice bath. Next, the DNA and proteins were transferred from the gel to a positively charged nylon membrane, fixed, blocked, washed and finally stained using solutions of an ECL Western blotting kit (GE Healthcare), and images were captured with a FlourChemQ system (Alpha Innotech).

### LC‐MS/MS analysis of ScCsoR

The purified ScCsoR was reacted with 10‐fold (molar ratio) of hydrogen polysulfide (HS_n_H, *n* ≥ 2) or DTT for 20 min at room temperature. The reacted protein was treated with a denaturing buffer (0.5 M Tri‐HCl, 2.75 mM EDTA, 6 M guanadine‐HCl, pH 8.0) containing 1 M iodoacetamide (IAM) for 1 h, and the sample was subsequently digested with trypsin (1:25, w/w) at 37 °C for 4 h. LC‐MS/MS analysis was conducted following a previously reported protocol (Li *et al*., 2017b). The Prominence nano‐LC system (Shimadzu) equipped with a custom‐made silica column (75 μm × 15 cm) packed with 3‐μm Reprosil‐Pur 120 C18‐AQ was used for the analysis. For the elution process, a total gradient of 100 min from 0% to 100% of solvent B (0.1% (v/v) formic acid in 98% (v/v) acetonitrile) at 300 nl min^−1^ was used; solvent A was 0.1% (v/v) formic acid in 2% (v/v) acetonitrile. The eluent was ionized and electrosprayed via LTQ‐Orbitrap Velos Pro CID mass spectrometer (Thermo Scientific); the run was performed in a data‐dependent acquisition mode with Xcalibur 2.2.0 software (Thermo Scientific). Full‐scan MS spectra (from 400 to 1800 *m/z*) were detected using the Orbitrap at a resolution of 60,000 at 400 *m/z*.

### Bioinformatics analysis

ScPDO and ScCsoR were used as queries to search homologues from all the sequenced Streptomyces genomes mentioned in the website, https://patricbrc.org. The selection criteria used were E‐value < 1e^‐60^, identity ≥ 35% and coverage ≥ 70%. ClustalW was used for alignment of these candidates, and a condensed neighbour‐joining tree was further built with bootstrap replications at 1000 by using the p‐distance method, uniform rates and pairwise deletion with a cut‐off at 50% by using the MEGA 7.0 software (Kumar *et al*., 2016).

## Conflict of interest

The authors declare no conflict of interest.

## Supporting information


**Fig. S1.** Amino acid sequence alignment analysis of ScPDO and known type III PDOs. The reported type III PDOs usually contain PDO and rhodanese domains. The representative type III PDOs amino acid sequences of *Zunongwangia profunda* SM‐A87 (ZpPDOIII, ADF52140.1), *Staphylococcus aureus* (SaPDOIII, WP_000465474.1) and *Bacillus cereus* ATCC 10876 (BcPdoIII, EEK49737.1) were downloaded from NCBI and were compared with ScPDO by using ClustalW. The results show that ScPDO is a typical type III PDOs, which is consistent with a previous report (Xia *et al*., 2017).
**Fig. S2.** SDS‐PAGE analysis of recombinant ScPDO. Lane 1 is the ladder (kDa), Lanes 2‐4 are ScPDO with different concentrations. The theoretical MW of His‐tag‐ScPDO is about 49 kDa.
**Fig. S3.** MS^2^ data of peptide 1, which was from DTT‐treated ScCsoR.
**Fig. S4.** MS^2^ data of peptide 2, which was from DTT‐treated ScCsoR.
**Fig. S5.** MS^2^ data of peptide 3, which was only detected from polysulfide‐treated ScCsoR.
**Fig. S6.** EMSA assay of ScCsoR‐C37S, ScCsoR‐C66S and ScCsoR‐C37S‐C66S mutants. The Cys‐to‐Ser mutation did not affect the ScCsoR binding activity to *Scpdo* promoter; however, it caused the loss of HS_n_H sensing activity and HS_n_H could not release ScCsoR mutants from *Scpdo* promoter. The promoter region of Scpdo (256 bp) was used as the DNA probe. 1 nM DNA probe was incubated with gradient concentration of ScCsoR mutants (0, 0.5, 1.0, 2.0, 4.0, 8.0, 8.0, 8.0, 8.0 μM), different amounts of polysulfides (0, 1, 2, 3 mM) was added to the reaction system (with equal amounts of ScCsoR mutants‐8.0 μM). The black arrow indicates the freedom DNA probe, the red arrow indicates the shifted DNA probe. These results show that the cysteines on ScCsoR play an important role for sulfane sulfur sensing, and the two cysteine are indispensable.
**Fig. S7.** The intergenic region of *SccsoR* and *Scrhod* promoters. The starting codon is shown in bold black, binding site is shown in red font. The number below is the length (bp) between the starting codon and binding site.
**Fig. S8.** *Sco0622* and *sco0623* expression was not controlled by ScCsoR. (A) Co‐transcription analysis of *Scpdo*‐*tauE*, *rhod*‐*sco0622* and *sco0622*‐*23*. Genomic DNA, mRNA, and cDNA as templates for lanes 1, 2, and 3, respectively. (B) EMSA analysis of ScCsoR binding activity to the promoter region of *sco0622* (242bp), 1 nM DNA probe was incubated with different amounts of ScCsoR (0, 2.2, 8.8, 15.4, 22.2 μM), the black arrow indicates the unbounded DNA probe. (C) RT‐qPCR analysis of the transcriptional level of *sco0622* in wt and Δ*SccsoR* strains. RNA was harvested at 36 h, 60 h, 84 h from mycelium grown in YBP solid medium. The *hrdB* gene was used as the internal control to normalize the mRNA level. Three independent measurements were carried out and error bars indicate the standard deviations.
**Table S1.** The distribution of *Scpdo‐rhod* in representative Streptomyces genomes.
**Table S2.** Strains and plasmids used in this study.
**Table S3.** Primers (5′→3′) used in this study.

## References

[mbt213637-bib-0001] Bai, C. , Zhang, Y. , Zhao, X. , Hu, Y. , Xiang, S. , Miao, J. , *et al* (2015) Exploiting a precise design of universal synthetic modular regulatory elements to unlock the microbial natural products in *Streptomyces* . Proc Natl Acad Sci USA 112: 12181–12186. 26374838 10.1073/pnas.1511027112 PMC4593075

[mbt213637-bib-0002] Barka, E.A. , Vatsa, P. , Sanchez, L. , Gaveau‐Vaillant, N. , Jacquard, C. , Meier‐Kolthoff, J.P. , *et al* (2016) Taxonomy, physiology, and natural products of Actinobacteria. Microbiol Mol Biol Rev 80: 1–43. 26609051 10.1128/MMBR.00019-15 PMC4711186

[mbt213637-bib-0003] Bentley, S.D. , Chater, K.F. , Cerdeno‐Tarraga, A.M. , Challis, G.L. , Thomson, N.R. , James, K.D. , *et al* (2002) Complete genome sequence of the model actinomycete *Streptomyces coelicolor* A3(2). Nature 417: 141–147. 12000953 10.1038/417141a

[mbt213637-bib-0004] Bibb, M.J. (2013) Understanding and manipulating antibiotic production in actinomycetes. Biochem Soc Trans 41: 1355–1364. 24256223 10.1042/BST20130214

[mbt213637-bib-0005] Blin, K. , Shaw, S. , Steinke, K. , Villebro, R. , Ziemert, N. , Lee, S.Y. , *et al* (2019) antiSMASH 5.0: updates to the secondary metabolite genome mining pipeline. Nucleic Acids Res 47: W81–W87. 31032519 10.1093/nar/gkz310 PMC6602434

[mbt213637-bib-0006] Bush, M.J. , Tschowri, N. , Schlimpert, S. , Flardh, K. , and Buttner, M.J. (2015) c‐di‐GMP signalling and the regulation of developmental transitions in streptomycetes. Nat Rev Microbiol 13: 749–760. 26499894 10.1038/nrmicro3546

[mbt213637-bib-0007] Bystrykh, L.V. , Fernandez‐Moreno, M.A. , Herrema, J.K. , Malpartida, F. , Hopwood, D.A. , and Dijkhuizen, L. (1996) Production of actinorhodin‐related “blue pigments” by *Streptomyces coelicolor* A3(2). J Bacteriol 178: 2238–2244. 8636024 10.1128/jb.178.8.2238-2244.1996 PMC177931

[mbt213637-bib-0008] Chang, F.M. , Coyne, H.J. , Cubillas, C. , Vinuesa, P. , Fang, X. , Ma, Z. , *et al* (2014) Cu(I)‐mediated allosteric switching in a copper‐sensing operon repressor (CsoR). J Biol Chem 289: 19204–19217. 24831014 10.1074/jbc.M114.556704 PMC4081955

[mbt213637-bib-0009] Chen, W. , Liu, C. , Peng, B. , Zhao, Y. , Pacheco, A. , and Xian, M. (2013) New fluorescent probes for sulfane sulfurs and the application in bioimaging. Chem Sci 4: 2892–2896. 23750317 10.1039/C3SC50754H PMC3673728

[mbt213637-bib-0010] Chen, S. , Zheng, G. , Zhu, H. , He, H. , Chen, L. , Zhang, W. , *et al* (2016) Roles of two‐component system AfsQ1/Q2 in regulating biosynthesis of the yellow‐pigmented coelimycin P2 in *Streptomyces coelicolor* . FEMS Microbiol Lett 363: fnw160 10.1093/femsle/fnw160. 27313101

[mbt213637-bib-0011] Coyne, H.J. 3rd , and Giedroc, D.P. (2013) Backbone resonance assignments of the homotetrameric (48 kD) copper sensor CsoR from *Geobacillus thermodenitrificans* in the apo‐ and Cu(I)‐bound states: insights into copper‐mediated allostery. Biomol NMR Assign 7: 279–283. 23001947 10.1007/s12104-012-9428-4 PMC3586942

[mbt213637-bib-0012] Festa, R.A. , Jones, M.B. , Butler‐Wu, S. , Sinsimer, D. , Gerads, R. , Bishai, W.R. , *et al* (2011) A novel copper‐responsive regulon in *Mycobacterium tuberculosis* . Mol Microbiol 79: 133–148. 21166899 10.1111/j.1365-2958.2010.07431.x PMC3052634

[mbt213637-bib-0013] Fukuto, J.M. , Ignarro, L.J. , Nagy, P. , Wink, D.A. , Kevil, C.G. , Feelisch, M. , *et al* (2018) Biological hydropersulfides and related polysulfides ‐ a new concept and perspective in redox biology. FEBS Lett 592: 2140–2152. 29754415 10.1002/1873-3468.13090 PMC6033183

[mbt213637-bib-0014] Giedroc, D.P. (2017) A new player in bacterial sulfide‐inducible transcriptional regulation. Mol Microbiol 105: 347–352. 28612383 10.1111/mmi.13726 PMC5548431

[mbt213637-bib-0015] Hildebrandt, T.M. , and Grieshaber, M.K. (2008) Three enzymatic activities catalyze the oxidation of sulfide to thiosulfate in mammalian and invertebrate mitochondria. FEBS J 275: 3352–3361. 18494801 10.1111/j.1742-4658.2008.06482.x

[mbt213637-bib-0016] Hopwood, D.A. (2007) Streptomyces in nature and medicine: the antibiotic makers. New York, NY, USA: Oxford University Press.

[mbt213637-bib-0017] Hou, N. , Yan, Z. , Fan, K. , Li, H. , Zhao, R. , Xia, Y. , *et al* (2019) OxyR senses sulfane sulfur and activates the genes for its removal in *Escherichia coli* . Redox Biol 26: 101293. 31421411 10.1016/j.redox.2019.101293 PMC6831875

[mbt213637-bib-0018] Huang, H. , Zheng, G. , Jiang, W. , Hu, H. , and Lu, Y. (2015) One‐step high‐efficiency CRISPR/Cas9‐mediated genome editing in *Streptomyces* . Acta Biochim Biophys Sin (Shanghai) 47: 231–243. 25739462 10.1093/abbs/gmv007

[mbt213637-bib-0019] Khosla, C. , McDaniel, R. , Ebert‐Khosla, S. , Torres, R. , Sherman, D.H. , Bibb, M.J. , and Hopwood, D.A. (1993) Genetic construction and functional analysis of hybrid polyketide synthases containing heterologous acyl carrier proteins. J Bacteriol 175: 2197–2204. 8468280 10.1128/jb.175.8.2197-2204.1993 PMC204504

[mbt213637-bib-0020] Kieser, T. , Bibb, M.J. , Buttner, M.J. , Chater, K.F. , and Hopwood, D.A. (2000) Practical streptomyces genetics. Norwich, UK: John Innes Foundation.

[mbt213637-bib-0021] Kumar, S. , Stecher, G. , and Tamura, K. (2016) MEGA7: Molecular evolutionary genetics analysis version 7.0 for bigger datasets. Mol Biol Evol 33: 1870–1874. 27004904 10.1093/molbev/msw054 PMC8210823

[mbt213637-bib-0022] Lau, N. , and Pluth, M.D. (2019) Reactive sulfur species (RSS): persulfides, polysulfides, potential, and problems. Curr Opin Chem Biol 49: 1–8. 30243097 10.1016/j.cbpa.2018.08.012

[mbt213637-bib-0023] Li, J.W. , and Vederas, J.C. (2009) Drug discovery and natural products: end of an era or an endless frontier? Science 325: 161–165. 19589993 10.1126/science.1168243

[mbt213637-bib-0024] Li, L. , Jiang, W. , and Lu, Y. (2017a) A novel two‐component system, GluR‐GluK, involved in glutamate sensing and uptake in *Streptomyces coelicolor* . J Bacteriol 199: e00097‐17. 28461451 10.1128/JB.00097-17 PMC5573080

[mbt213637-bib-0025] Li, H. , Li, J. , Lu, C. , Xia, Y. , Xin, Y. , Liu, H. , *et al* (2017b) FisR activates sigma(54) ‐dependent transcription of sulfide‐oxidizing genes in *Cupriavidus pinatubonensis* JMP134. Mol Microbiol 105: 373–384. 28612361 10.1111/mmi.13725

[mbt213637-bib-0026] de Lira, N.P.V. , Pauletti, B.A. , Marques, A.C. , Perez, C.A. , Caserta, R. , de Souza, A.A. , *et al* (2018) BigR is a sulfide sensor that regulates a sulfur transferase/dioxygenase required for aerobic respiration of plant bacteria under sulfide stress. Sci Rep 8: 3508. 29472641 10.1038/s41598-018-21974-x PMC5823870

[mbt213637-bib-0027] Liu, G. , Chater, K.F. , Chandra, G. , Niu, G. , and Tan, H. (2013) Molecular regulation of antibiotic biosynthesis in streptomyces. Microbiol Mol Biol Rev 77: 112–143. 23471619 10.1128/MMBR.00054-12 PMC3591988

[mbt213637-bib-0028] Liu, H. , Xin, Y. , and Xun, L. (2014) Distribution, diversity, and activities of sulfur dioxygenases in heterotrophic bacteria. Appl Environ Microbiol 80: 1799–1806. 24389926 10.1128/AEM.03281-13 PMC3957605

[mbt213637-bib-0029] Lu, T. , Zhu, Y. , Zhang, P. , Sheng, D. , Cao, G. , and Pang, X. (2018) SCO5351 is a pleiotropic factor that impacts secondary metabolism and morphological development in *Streptomyces coelicolor* . FEMS Microbiol Lett 365 10.1093/femsle/fny150 29931327

[mbt213637-bib-0030] Luebke, J.L. , Shen, J. , Bruce, K.E. , Kehl‐Fie, T.E. , Peng, H. , Skaar, E.P. , and Giedroc, D.P. (2014) The CsoR‐like sulfurtransferase repressor (CstR) is a persulfide sensor in *Staphylococcus aureus* . Mol Microbiol 94: 1343–1360. 25318663 10.1111/mmi.12835 PMC4264537

[mbt213637-bib-0031] Mak, S. , and Nodwell, J.R. (2017) Actinorhodin is a redox‐active antibiotic with a complex mode of action against Gram‐positive cells. Mol Microbiol 106: 597–613. 28906045 10.1111/mmi.13837

[mbt213637-bib-0032] Motl, N. , Skiba, M.A. , Kabil, O. , Smith, J.L. , and Banerjee, R. (2017) Structural and biochemical analyses indicate that a bacterial persulfide dioxygenase‐rhodanese fusion protein functions in sulfur assimilation. J Biol Chem 292: 14026–14038. 28684420 10.1074/jbc.M117.790170 PMC5572905

[mbt213637-bib-0033] Nett, M. , Ikeda, H. , and Moore, B.S. (2009) Genomic basis for natural product biosynthetic diversity in the actinomycetes. Nat Prod Rep 26: 1362–1384. 19844637 10.1039/b817069j PMC3063060

[mbt213637-bib-0034] Newton, G.L. , Buchmeier, N. , and Fahey, R.C. (2008) Biosynthesis and functions of mycothiol, the unique protective thiol of Actinobacteria. Microbiol Mol Biol Rev 72: 471–494. 18772286 10.1128/MMBR.00008-08 PMC2546866

[mbt213637-bib-0035] Peng, H. , Zhang, Y. , Palmer, L.D. , Kehl‐Fie, T.E. , Skaar, E.P. , Trinidad, J.C. , and Giedroc, D.P. (2017) Hydrogen sulfide and reactive sulfur species impact proteome S‐sulfhydration and global virulence regulation in *Staphylococcus aureus* . ACS Infect Dis 3: 744–755. 28850209 10.1021/acsinfecdis.7b00090 PMC5863038

[mbt213637-bib-0036] Ran, M. , Wang, T. , Shao, M. , Chen, Z. , Liu, H. , Xia, Y. , and Xun, L. (2019) Sensitive method for reliable quantification of sulfane sulfur in biological samples. Anal Chem 91: 11981–11986. 31436086 10.1021/acs.analchem.9b02875

[mbt213637-bib-0037] Robertsen, H.L. , and Musiol‐Kroll, E.M. (2019) Actinomycete‐derived polyketides as a source of antibiotics and lead structures for the development of new antimicrobial drugs. Antibiotics 8: 157. 10.3390/antibiotics8040157 PMC6963833 31547063

[mbt213637-bib-0038] Shen, B. (2015) A new golden age of natural products drug discovery. Cell 163: 1297–1300. 26638061 10.1016/j.cell.2015.11.031 PMC5070666

[mbt213637-bib-0039] Shen, J. , Keithly, M.E. , Armstrong, R.N. , Higgins, K.A. , Edmonds, K.A. , and Giedroc, D.P. (2015) *Staphylococcus aureus* CstB is a novel multidomain persulfide dioxygenase‐sulfurtransferase involved in hydrogen sulfide detoxification. Biochemistry 54: 4542–4554. 26177047 10.1021/acs.biochem.5b00584 PMC4874178

[mbt213637-bib-0040] Shi, X. , Festa, R.A. , Ioerger, T.R. , Butler‐Wu, S. , Sacchettini, J.C. , Darwin, K.H. , and Samanovic, M.I. (2014) The copper‐responsive RicR Regulon contributes to *Mycobacterium tuberculosis* virulence. MBio 5: e00876‐13. 24549843 10.1128/mBio.00876-13 PMC3944814

[mbt213637-bib-0041] Shimizu, T. , and Masuda, S. (2020) Persulphide‐responsive transcriptional regulation and metabolism in bacteria. J Bio chem 167: 125–132. 10.1093/jb/mvz063 31385583

[mbt213637-bib-0042] Shimizu, T. , Shen, J. , Fang, M. , Zhang, Y. , Hori, K. , Trinidad, J.C. , *et al* (2017) Sulfide‐responsive transcriptional repressor SqrR functions as a master regulator of sulfide‐dependent photosynthesis. Proc Natl Acad Sci USA 114: 2355–2360. 28196888 10.1073/pnas.1614133114 PMC5338557

[mbt213637-bib-0043] Strohl, W.R. , and Connors, N.C. (1992) Significance of anthraquinone formation resulting from the cloning of actinorhodin genes in heterologous streptomycetes. Mol Microbiol 6: 147–152. 1545701 10.1111/j.1365-2958.1992.tb01995.x

[mbt213637-bib-0044] Weinitschke, S. , Denger, K. , Cook, A.M. , and Smits, T.H. (2007) The DUF81 protein TauE in Cupriavidus necator H16, a sulfite exporter in the metabolism of C2 sulfonates. Microbiology 153: 3055–3060. 17768248 10.1099/mic.0.2007/009845-0

[mbt213637-bib-0045] Wright, G.D. (2017) Opportunities for natural products in 21(st) century antibiotic discovery. Nat Prod Rep 34: 694–701. 28569300 10.1039/c7np00019g

[mbt213637-bib-0050] Xia, Y. , Chu, W. , Qi, Q. , and Xun, L. (2015) New insights into the QuikChangeTM process guide the use of Phusion DNA polymerase for site‐directed mutagenesis. Nucleic Acids Res, 43: e12. 25399421 10.1093/nar/gku1189 PMC4333370

[mbt213637-bib-0046] Xia, Y. , Lu, C. , Hou, N. , Xin, Y. , Liu, J. , Liu, H. , and Xun, L. (2017) Sulfide production and oxidation by heterotrophic bacteria under aerobic conditions. ISME J 11: 2754–2766. 28777380 10.1038/ismej.2017.125 PMC5702731

[mbt213637-bib-0047] Xin, Y. , Liu, H. , Cui, F. , Liu, H. , and Xun, L. (2016) Recombinant Escherichia coli with sulfide:quinone oxidoreductase and persulfide dioxygenase rapidly oxidises sulfide to sulfite and thiosulfate via a new pathway. Environ Microbiol 18: 5123–5136. 27573649 10.1111/1462-2920.13511

[mbt213637-bib-0048] Xu, Z. , Wang, Y. , Chater, K.F. , Ou, H.Y. , Xu, H.H. , Deng, Z. , and Tao, M. (2017) Large‐scale transposition mutagenesis of *Streptomyces coelicolor* identifies hundreds of genes influencing antibiotic biosynthesis. Appl Environ Microbiol 83: e02889‐16. 28062460 10.1128/AEM.02889-16 PMC5335527

[mbt213637-bib-0049] Xu, Z. , Li, Y. , Wang, Y. , Deng, Z. , and Tao, M. (2019) Genome‐wide mutagenesis links multiple metabolic pathways with actinorhodin production in *Streptomyces coelicolor* . Appl Environ Microbiol 85: e03005‐18. 30709825 10.1128/AEM.03005-18 PMC6585502

