# Supplementary material for: Sulfane sulfur‐activated actinorhodin production and sporulation is maintained by a natural gene circuit in Streptomyces coelicolor
Source: Microb Biotechnol. 2020 Aug 9;13(6):1917–32. doi: 10.1111/1751-7915.13637 (PMC7533328; doi:10.1111/1751-7915.13637)
Supplement: Supplementary file 1 — Fig. S1. Amino acid sequence alignment analysis of ScPDO and known type III PDOs. The reported type III PDOs usually contain PDO and rhodanese domains. The representative type III PDOs amino acid sequences of Zunongwangia profunda SM‐A87 (ZpPDOIII, ADF52140.1), Staphylococcus aureus (SaPDOIII, WP_000465474.1) and Bacillus cereus ATCC 10876 (BcPdoIII, EEK49737.1) were downloaded from NCBI and were compared with ScPDO by using ClustalW. The results show that ScPDO is a typical type III PDOs, which is consistent with a previous report (Xia et al., 2017). Fig. S2. SDS‐PAGE analysis of recombinant ScPDO. Lane 1 is the ladder (kDa), Lanes 2‐4 are ScPDO with different concentrations. The theoretical MW of His‐tag‐ScPDO is about 49 kDa. Fig. S3. MS2 data of peptide 1, which was from DTT‐treated ScCsoR. Fig. S4. MS2 data of peptide 2, which was from DTT‐treated ScCsoR. Fig. S5. MS2 data of peptide 3, which was only detected from polysulfide‐treated ScCsoR. Fig. S6. EMSA assay of ScCsoR‐C37S, ScCsoR‐C66S and ScCsoR‐C37S‐C66S mutants. The Cys‐to‐Ser mutation did not affect the ScCsoR binding activity to Scpdo promoter; however, it caused the loss of HSnH sensing activity and HSnH could not release ScCsoR mutants from Scpdo promoter. The promoter region of Scpdo (256 bp) was used as the DNA probe. 1 nM DNA probe was incubated with gradient concentration of ScCsoR mutants (0, 0.5, 1.0, 2.0, 4.0, 8.0, 8.0, 8.0, 8.0 μM), different amounts of polysulfides (0, 1, 2, 3 mM) was added to the reaction system (with equal amounts of ScCsoR mutants‐8.0 μM). The black arrow indicates the freedom DNA probe, the red arrow indicates the shifted DNA probe. These results show that the cysteines on ScCsoR play an important role for sulfane sulfur sensing, and the two cysteine are indispensable. Fig. S7. The intergenic region of SccsoR and Scrhod promoters. The starting codon is shown in bold black, binding site is shown in red font. The number below is the length (bp) between the starting codon a [file MBT2-13-1917-s001.docx]

**Supporting information**

Sulfane sulfur-activated actinorhodin production and sporulation is maintained by a natural gene circuit in *Streptomyces coelicolor*

Ting Lu^1^, Qun Cao^1^, Xiuhua Pang^1^, Yongzhen Xia^1^, Luying Xun^1,2^, Huaiwei Liu^1^*

Content:

Fig. S1—Fig. S9

Table S1—Table S3

**
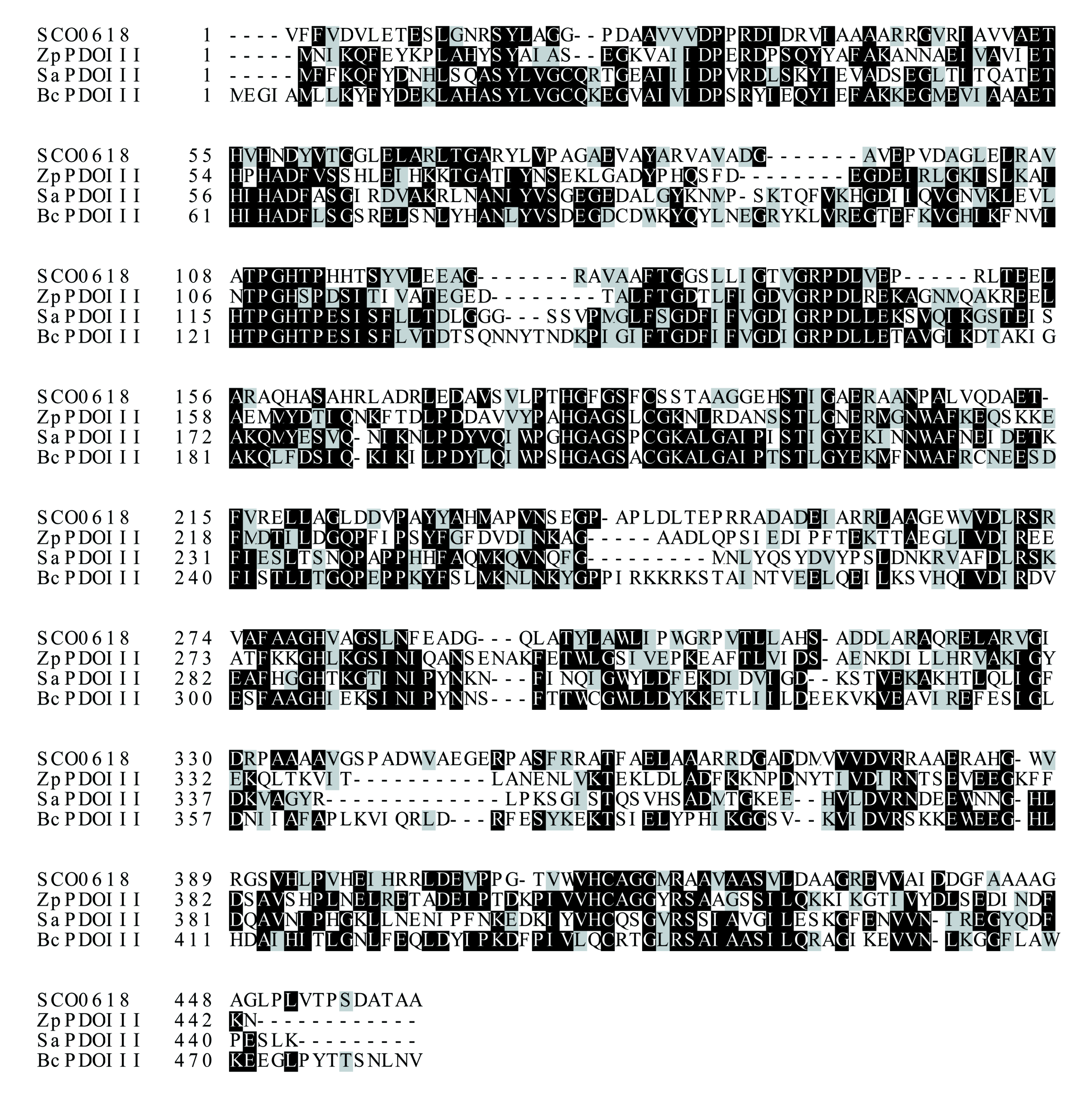
**

**Figure S1. Amino acid sequence alignment analysis of ScPDO and known type III PDOs.** The reported type III PDOs usually contain PDO and rhodanese domains. The representative type III PDOs amino acid sequences of *Zunongwangia profunda* SM-A87 (ZpPDOIII, ADF52140.1), *Staphylococcus aureus* (SaPDOIII, WP_000465474.1) and *Bacillus cereus* ATCC 10876 (BcPdoIII, EEK49737.1) were downloaded from NCBI and were compared with ScPDO by using ClustalW. The results show that ScPDO is a typical type III PDOs, which is consistent with a previous report (Xia, et al., 2017).

**
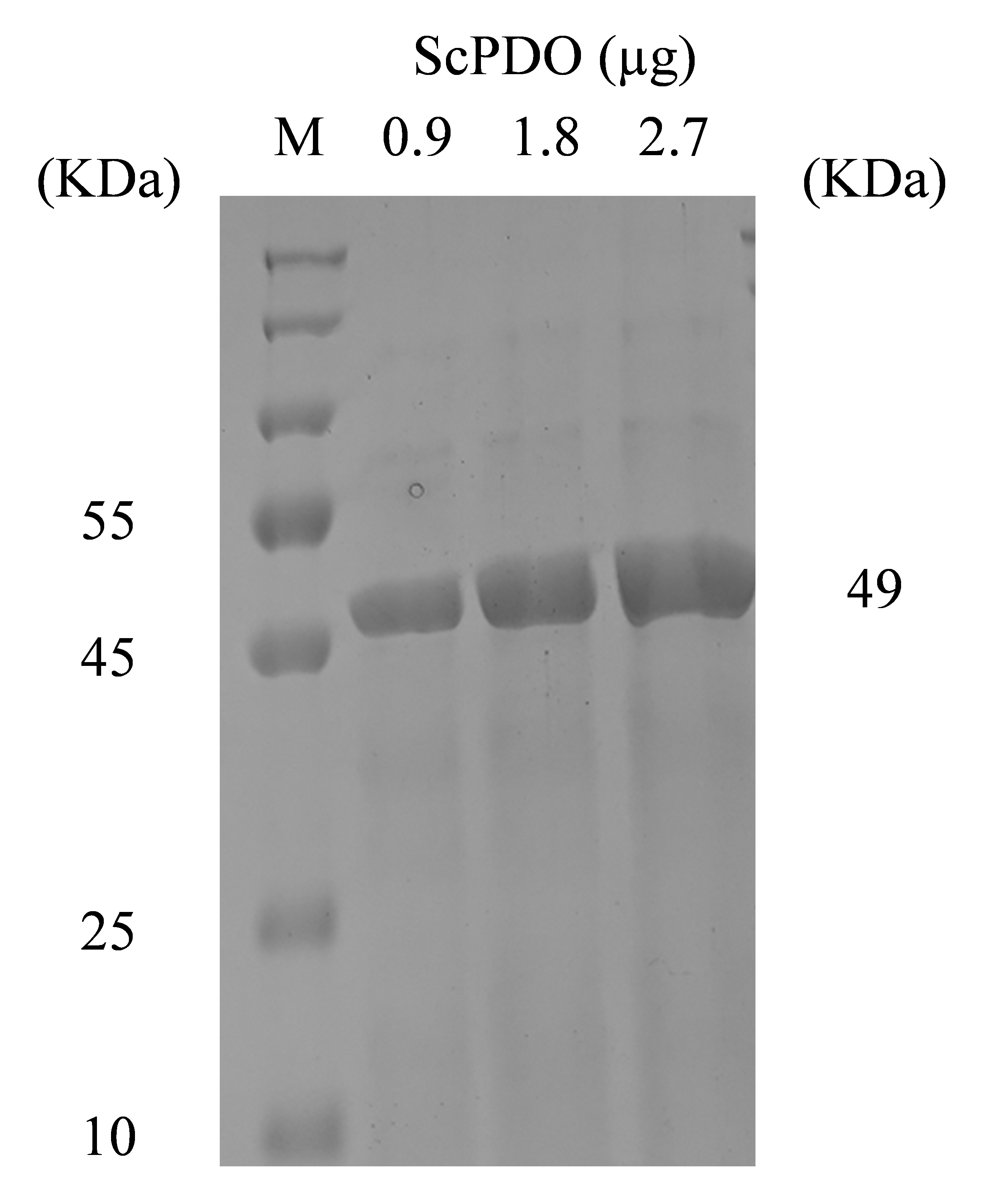
**

**Figure S2. SDS-PAGE analysis of recombinant ScPDO.** Lane 1 is the ladder (kDa), Lanes 2-4 are ScPDO with different concentrations. The theoretical MW of His-tag-ScPDO is about 49 kDa.


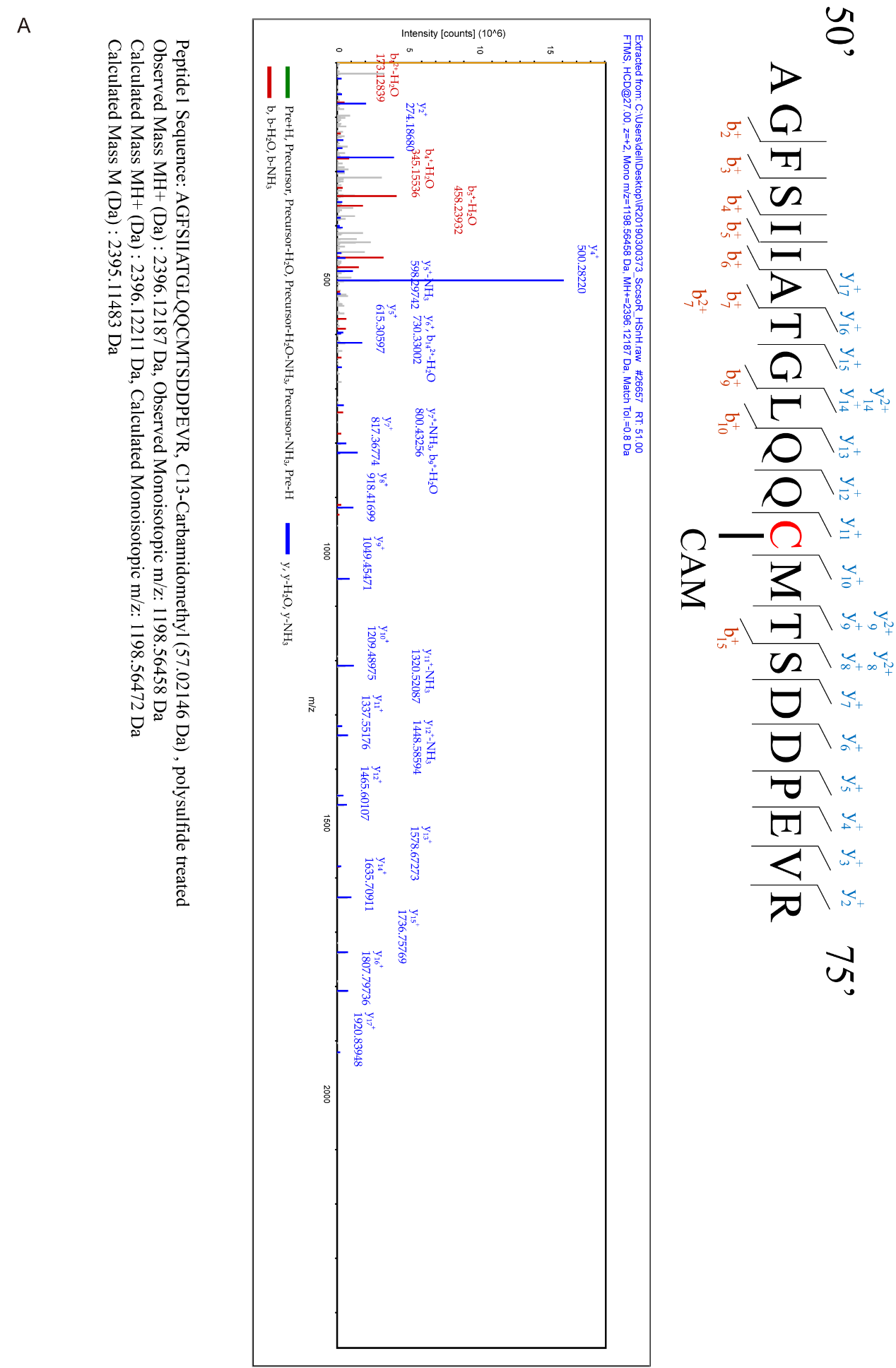


**Figure S3. MS^2^ data of peptide 1, which was from DTT-treated ScCsoR.**


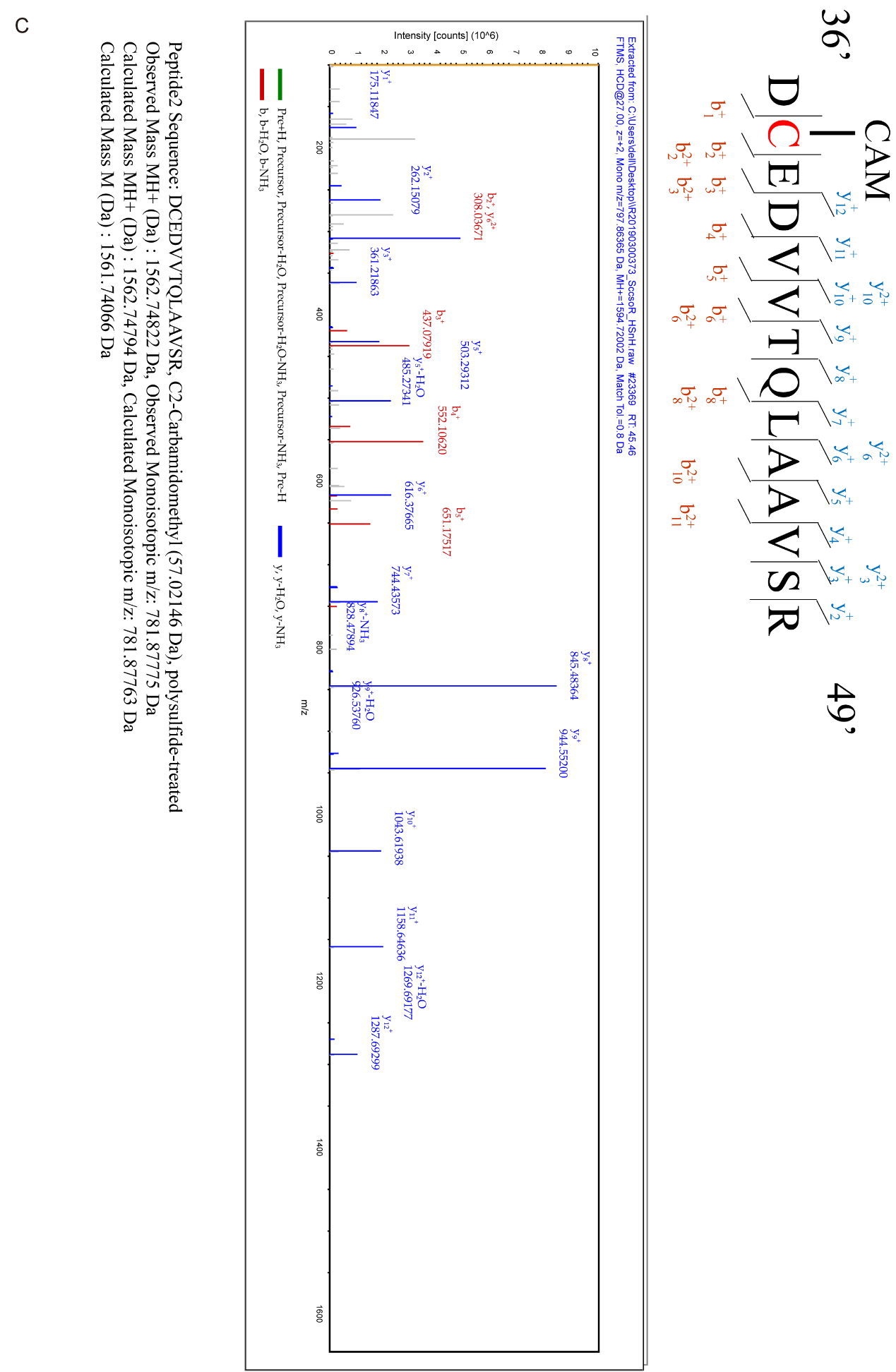


**Figure S4. MS^2^ data of peptide 2, which was from DTT-treated ScCsoR.**


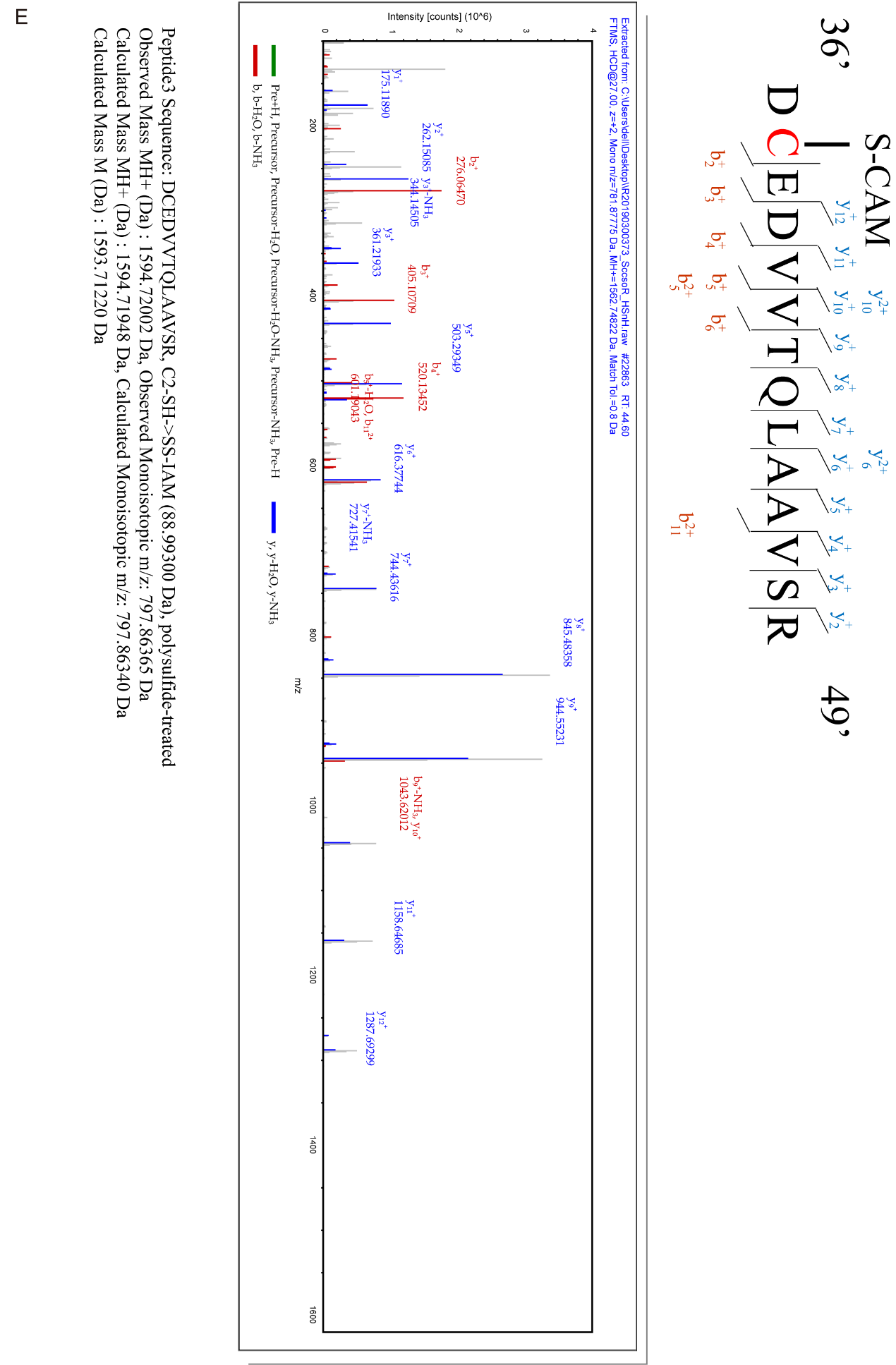


**Figure S5. MS^2^ data of peptide 3, which was only detected from polysulfide-treated ScCsoR.**


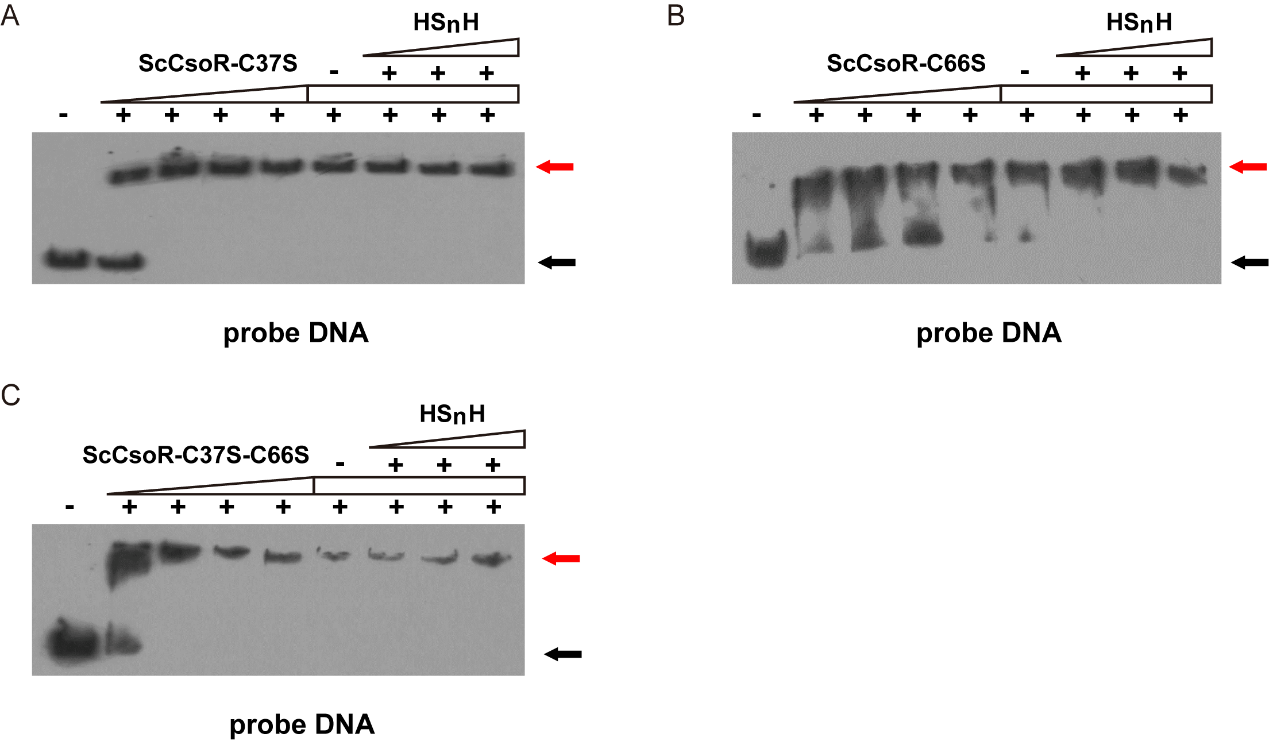


**Figure S6. EMSA assay of ScCsoR-C37S, ScCsoR-C66S and ScCsoR-C37S-C66S mutants.** The Cys-to-Ser mutation did not affect the ScCsoR binding activity to *Scpdo* promoter; however, it caused the loss of HS_n_H sensing activity and HS_n_H could not release ScCsoR mutants from *Scpdo* promoter. The promoter region of Scpdo (256bp) was used as the DNA probe. 1 nM DNA probe was incubated with gradient concentration of ScCsoR mutants (0, 0.5, 1.0, 2.0, 4.0, 8.0, 8.0, 8.0, 8.0 μM), different amounts of polysulfides (0, 1, 2, 3 mM) was added to the reaction system (with equal amounts of ScCsoR mutants-8.0 μM). The black arrow indicates the freedom DNA probe, the red arrow indicates the shifted DNA probe. These results show that the cysteines on ScCsoR play an important role for sulfane sulfur sensing, and the two cysteine are indispensable.


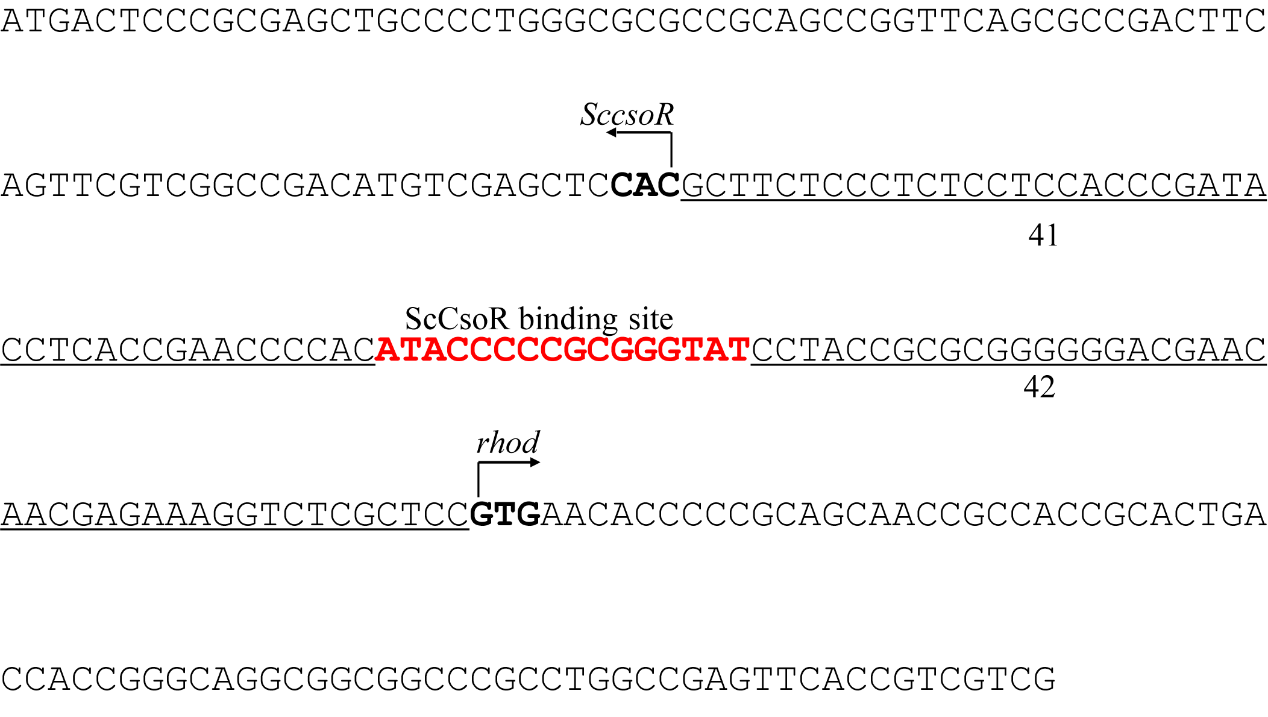


**Figure S7. The intergenic region of *SccsoR* and *Scrhod* promoters.**

The starting codon is shown in bold black, binding site is shown in red font. The number below is the length (bp) between the starting codon and binding site.


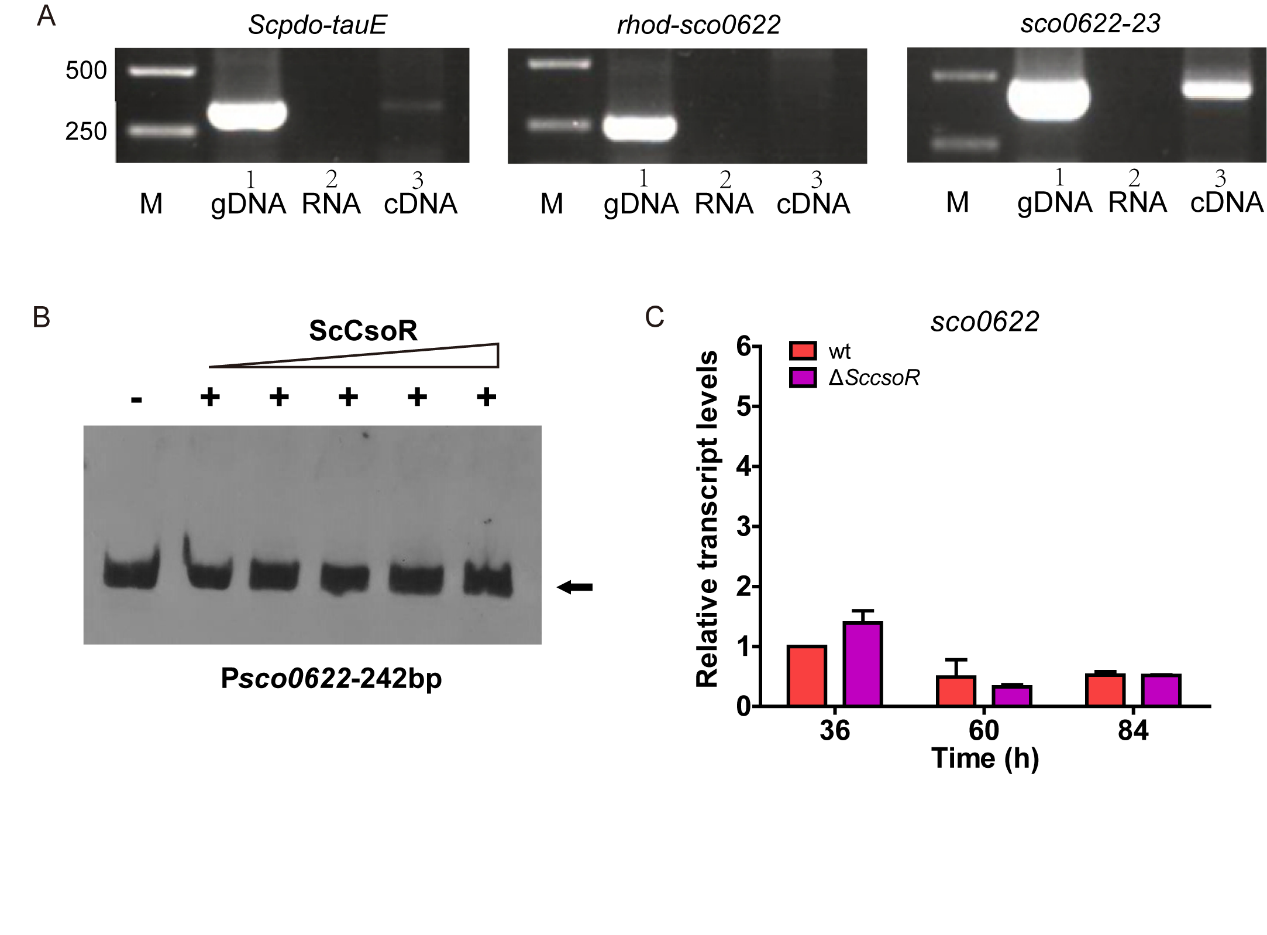


**Figure S8. *Sco0622* and *sco0623* expression was not controlled by ScCsoR.**

(A) Co-transcription analysis of *Scpdo*-*tauE*, *rhod*-*sco0622* and *sco0622*-*23*. Genomic DNA, mRNA, and cDNA as templates for lanes 1, 2, and 3, respectively.

(B) EMSA analysis of ScCsoR binding activity to the promoter region of *sco0622* (242bp), 1 nM DNA probe was incubated with different amounts of ScCsoR (0, 2.2, 8.8, 15.4, 22.2 μM), the black arrow indicates the unbounded DNA probe.

(C) RT-qPCR analysis of the transcriptional level of *sco0622* in wt and *ΔSccsoR* strains. RNA was harvested at 36 h, 60 h, 84 h from mycelium grown in YBP solid medium. The *hrdB* gene was used as the internal control to normalize the mRNA level. Three independent measurements were carried out and error bars indicate the standard deviations.


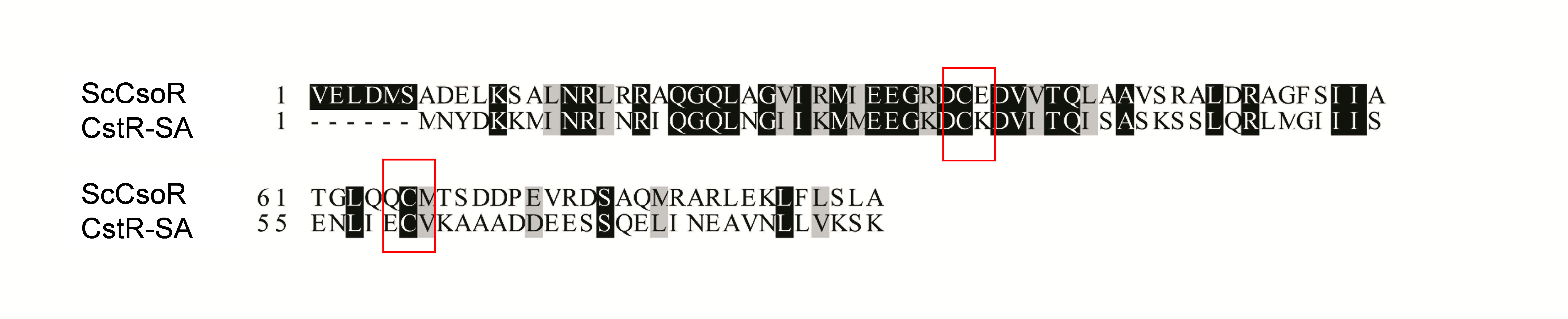


**Figure S9. Amino acid sequence alignment of ScCsoR and CstR.** The bioinformatics comparison showed that ScCsoR and CstR both have two conserved cysteines and 46% identities. The red square indicates the position of conserved cysteine.

**Table S1. The distribution of *Scpdo-rhod* in representative Streptomyces genomes.**

| Strain | Famous (Polyketides) Antibiotics | References |
| --- | --- | --- |
| *Streptomyces fradiae strain ATCC 19609* | Tylosin A, Neomycin | (Dulmage, 1953;Cundliffe, et al., 2001) |
| *Streptomyces rimosus subsp. rimosus strain NRRL WC-3869* | Oxytetracycline/Terracycline, Tylosin | (Mindlin, et al., 1961;Pape and Brillinger, 1973;Yin, et al., 2015) |
| *Streptomyces pristinaespiralis strain HCCB 10218* | Pristinamycin/Pyostacine | (Mast, et al., 2015) |
| *Saccharopolyspora erythraea NRRL 2338 strain DSM 40517* | Erythromycin A | (Oliynyk, et al., 2007) |
| *Streptomyces albulus strain B-3066* | Tetrafungin/Nystatin | (Veiga, et al., 1983) |
| *Streptomyces fungicidicus strain TXX3120* | Nystatin | (Matsuoka, 1960) |
| *Streptomyces lydicus strain 103* | Pimaricin/Natamycin, streptolydigin | (Sui, et al., 2009;Jia, et al., 2017) |
| *Streptomyces albus subsp. albus strain NRRL B-2513* | Salinomycin /Procoxacin,  Antimycin, Candicidin | (Jiang, et al., 2012;Olano, et al., 2014;McLean, et al., 2016) |
| *Streptomyces antibioticus strain DSM 41481* | Oleandomycin/Erythromycin, Kitamycin C/Antimycin | (Mendez, et al., 2002;Wang, et al., 2017) |
| *Streptomyces natalensis ATCC 27448* | Pimaricin/Natamycin | (Aparicio, et al., 2000;Elsayed, et al., 2019) |
| *Streptomyces hygroscopicus subsp. jinggangensis 5008* | validamycin A | (Zhou, et al., 2014) |
| *Streptomyces lincolnensis strain NRRL 2936* | Lincomycin | (Peschke, et al., 1995;Imai, et al., 2015) |
| *Streptomyces griseus strain S4-7* | Streptomycin | (Takano, et al., 2016) |
| *Streptomyces kanamyceticus strain ATCC 12853* | Kanamycin | (Kharel, et al., 2004) |
| *Streptomyces bingchenggensis BCW-1* | Milbemycins, Nanchangmycin | (Wang, et al., 2010;Zhang, et al., 2013) |

**Table S2. Strains and plasmids used in this study**

| Strain or plasmid | Description | Reference |
| --- | --- | --- |
| ***S.coelicolor* strains** | | |
| M145 | Wild type strain that produces multiple antibiotics, spc1^-^ and spc2^-^ | (Kieser, et al., 2000) |
| Δ*Scpdo* | M145 strain with disruption of *Scpdo* gene | This study |
| Δ*Scpdo*:: *Scpdo* | Δ*Scpdo* complemented with pCom-*Scpdo*, Hyg^R^ | This study |
| Vector-Δ*Scpdo* | Δ*Scpdo* complemented with pMS82 vector, Hyg^R^ | This study |
| Δ*SccsoR* | M145 strain with disruption of *SccsoR* gene, Apr^R^ | This study |
| Δ*SccsoR*::*SccsoR* | Δ*SccsoR* complemented with pCom-*SccsoR*, Apr^R^, Hyg^R^ | This study |
| Vector-Δ*SccsoR* | Δ*SccsoR* complemented with pMS82 vector, Apr^R^, Hyg^R^ | This study |
| ***E. coli* strains** | | |
| DH5α | Cloning strain | Invitrogen |
| BL21 (DE3) | Host used for protein expression | Invitrogen |
| ET12567(pUZ8002) | Strain used for conjugation, with plasmid pUZ8002, Kana^R^,Cm^R^ | (Kieser, et al., 2000) |
| **Plasmids** | | |
| pMD18-T | Cloning vector, Amp^R^ | Takara |
| pMD-*Scpdo* RACE | pMD18-T derivative for *Scpdo* 5-RACE sequencing | This study |
| pJTU1278 | The shuttle vector for gene mutation, Amp^R^ | (He, et al., 2010) |
| pJTU-mu *SccsoR* | pJTU1278 derivative for *SccsoR* disruption, Apr^R^, Amp^R^ | This study |
| pIJ773 | Template for apramycin resistance gene, Apr^R^ | (Yu, et al., 2012) |
| pKCcas9dO | The vector for gene mutation based on pSG5, Apr^R^ | (Huang, et al., 2015) |
| pKCcas9dO-*Scpdo* | pKCcas9dO derivative for *Scpdo* disruption, Apr^R^ | This study |
| pET-15b | Expression vector for proteins, Amp^R^ | Novagen |
| pEX-ScPDO | *Scpdo* expression vector, Amp^R^ | This study |
| pEX-ScCsoR | *SccsoR* expression vector, Amp^R^ | This study |
| pEX-ScCsoR-C37S | *SccsoR-C37S* expression vector, Amp^R^ | This study |
| pEX-ScCsoR-C66S | *SccsoR-C66S* expression vector, Amp^R^ | This study |
| pEX-ScCsoR-C37S-C66S | *SccsoR-C37S-C66S* expression vector, Amp^R^ | This study |
| pMS82 | Integrative vector for gene complementation, Hyg^R^ | (Gregory, et al., 2003) |
| pCom0618 | pMS82 derivative for *Scpdo* gene complementation, Hyg^R^ | This study |
| pCom-*SccsoR* | pMS82 derivative for *SccsoR* gene complementation, Hyg^R^ | This study |

**Table S3. Primers (5′→3′) used in this study**

| Primer | Sequence (5'-3') | Function |
| --- | --- | --- |
| Scpdo tar-up-f | AGTGGCACCGAGTCGGTGCTTTTTTTGAGGAGATGGGTGTGGACCAGGTTGAGG | Amplifying up region for *Scpdo* disruption |
| Scpdo tar-up-r | TACGAAGTGTGGTGCGGGGTGTGGC |  |
| Scpdo tar-down-f | ACCCCGCACCACACTTCGTACAACTTCGAGGCCGACGGACAGCTCGC | Amplifying down region for *Scpdo* disruption |
| Scpdo tar-down-r | GCATGCCTGCAGGTCGACGATAAAGCTTCAGCAGCAGGGTGAGGTCCAGGTGT |  |
| Scpdo tar-sgRNA-f | GGGATCCTCTAGAGATTCCCACTAGTACCGGCGGTTCGCTGCTGATGTTTTAGAGCTAGAAATA | Amplifying sgRNA for  *Scpdo* disruption |
| Scpdo tar-sgRNA-r | CTCAAAAAAAGCACCGACTCGGTGCCACT |  |
| Scpdo ver-f | CCACACCCCGCACCACACTTCGTAC | Verification of *Scpdo* disruption |
| Scpdo ver-r | GCCGTACGTCCACGACCACCATGTC |  |
| Scpdo com-f1 | GAGAACCTAGGATCCAAGCTTtgttcacattcgaaccgtctctgct | Complementary *Scpdo* by pMS82- for PCR kasop* promoter |
| Scpdo com-r1 | AGGACGTCGACAAAGAACACTAGATGTCTCCTTACTTAGA |  |
| Scpdo com-f2 | GTGTTCTTTGTCGACGTCCTGGAGACCGAGTCACTGGGCA | Complementary *Scpdo* by pMS82- for PCR *Scpdo* ORF |
| Scpdo com-r2 | ACCATGCATAGATCTAAGCTTTCATGCGGCGGTGGCGTCCG |  |
| ScCscoR left-f | ACCCGGGGATCCTCTAGAGATTCCCAAGCTTGTGACGCTGCTGGCGCACAGTG | Amplifying left arm for *SccsoR* disruption |
| ScCscoR left-r | CCAGTCGATTGGCTGAGCTCATGACCAGTGACGACCCCGAGG |  |
| ScCscoR right-f | TCATAGCACGATCAACGGCACTGTTGACCACGTCCTCGCAGTCCCG | Amplifying right arm for  *SccsoR* disruption |
| ScCscoR right-r | GCATGCCTGCAGGTCGACGATACCCTCTAGACTCGTGCTGCTGTTCCGCCTCG |  |
| ScCscoR apra-f | GAGCTCAGCCAATCGACTGGCGA | Amplifying apramycin for *SccsoR* disruption |
| ScCscoR apra-r | ACAGTGCCGTTGATCGTGCTATGA |  |
| ScCscoR ver-f | GAGAGGAAGAGCTTCTCCAGCCTGG | Verification of *SccsoR* disruption |
| ScCscoR ver-r | GGTATCGGGTGGAGGAGAGGGAGAA |  |
| ScCscoR com-f | GAGAACCTAGGATCCAAGCTTCACGAAACCGGCGGCGACGAGCGAC | Complementary *SccsoR* by pMS82 |
| ScCscoR com-r | ACCATGCATAGATCTAAGCTTCTCACCCTGCTGCTGCCGTTCCTGG |  |
| co-Scpdo-tauE-f | GTGCACCTGCCCGTCCACGAGATCC | Co-transcription test of *Scpdo* and *tauE* |
| co-Scpdo-tauE-r | GGGTGAAGCCGAGCAGGTAGACGAG |  |
| co-Scrhod-0622-f | ACGTCGCCTCCTTCGAGGAGACGCT | Co-transcription test of *Scrhod* and *sco0622* |
| co-Scrhod-0622-r | GGTGACCTTCTCCGCCCCGTGTTCG |  |
| co-Scsco0622-23-f | GGCGGAACAGCAGCACGAGCTGTGG | Co-transcription test of *sco0622* and *sco0623* |
| co-Scsco0622-23-r | GAGTATCTGCCGGTGGTTCATCGAG |  |
| hrdB-realtime-f | CGCCGAGTCCGTCTCTGTCA | Real-time PCR for reference gene |
| hrdB-realtime-r | GCTCTGCGGCACTGACCATC |  |
| Scpdo-realtime-f | GCGGCGAGCACAGCACGAT | Real-time PCR for *Scpdo* |
| Scpdo-realtime-r | TGGGCGTAGTAGGCGGGCA |  |
| Scpdo-realtime-f | GGCCGCGAAGGTCTCCGCCGGGAC | Real-time PCR for *tauE* |
| Scpdo-realtime-r | TCACAGTACCGCCGACACGCCCAT |  |
| ScCscoR-realtime-f | ACTGCGAGGACGTGGTCACC | Real-time PCR for *csoR* |
| ScCscoR-realtime-r | GCCCGCATCTGTGCCGAGT |  |
| rhod-realtime-f | ACTGGTCTTCTCCGGAGTGACCAA | Real-time PCR for *rhod* |
| rhod-realtime-r | CAGGCGGCCAGTCGGGCCAGCG |  |
| sco0622-realtime-f | TCTGCGGGAGCGGAAGAAGCGG | Real-time PCR for *sco0622* |
| sco0622-realtime-r | TTCTCCGCCCCGTGTTCGGC |  |
| ScPDO His15bF | GTGCCGCGCGGCAGCCATATGGTGTTCTTTGTCGACGTCCTGGAGA | ScPDO recombinant expression |
| ScPDO His15bR | GTTAGCAGCCGGATCCTCGAGTCATGCGGCGGTGGCGTCCGAGGGC |  |
| ScCsoR His15bF | GTGCCGCGCGGCAGCCATATGGTGGAGCTCGACATGTCGGCCG | ScCsoR-recombinant expression |
| ScCsoR His15bR | GTTAGCAGCCGGATCCTCGAGTCAGGCCAGGGAGAGGAAGAGC |  |
| ScCsoR C37SHis15bF1 | AGGACGTGGTCACCCAGCTCGCC | ScCsoRC37S-recombinant expression |
| ScCsoR C37SHis15bR1 | TGGGTGACCACGTCCTCGGAGTCC |  |
| ScCsoR C66SHis15bF2 | ACTGCAGCAGTCCATGACCAGTGACGA | ScCsoRC66S-recombinant expression |
| ScCsoR C66SHis15bR2 | GTCATGGACTGCTGCAGTCCCGTG |  |
| Scpdo EMSA-f | GTACCCCAACTTCGGCGTCGTACACCG | 256bp fragment for EMSA |
| Scpdo EMSA-r | GCCAGATAGCTGCGGTTGCCCAGTGAC |  |
| ScCscoR-rhod EMSA-f | ATGACTCCCGCGAGCTGCCCCTG | 261bp fragment for EMSA |
| ScCscoR-rhod EMSA-r | CGACGACGGTGAACTCGGCCAGG |  |
| sco0622 EMSA-f | CGAGGAGACGCTGGCCCGACTGG | 242bp fragment for EMSA |
| sco0622 EMSA-r | CGGTGACCTTCTCCGCCCCGTGT |  |
| p-e1-f | GTACCCCAACTTCGGCGTCGTACACCG | 106bp fragment for EMSA |
| p-e1-r | TCTGCTCACGCCGCCCCCGCGCCGT |  |
| p-e2-f | ACGGCGCGGGGGCGGCGTGAGCAGA | 110bp fragment for EMSA |
| p-e2-r | TCCTTCCGGAATTACCCCCT |  |
| p-e3-f | GCACACCCTCTCAATACCC | 108bp fragment for EMSA |
| p-e3-r | AGGACGTCGACAAAGAACACGGC |  |
| p-e4-f | ACGGTGGCCGGAAGAGATACCCC | 108bp fragment for EMSA |
| p-e4-r | GCCAGATAGCTGCGGTTGCCCAGTGAC |  |
| Scpdo EMSA79bp-f 5-Biotin | CCTGGGTATATCTGTTACGGTGGCCGGAAGAGATACCCCAGGGGGTAATTCCGGAAGGAGAGCGTGCCGTGTTCTTTGT | 79bp fragment for EMSA |
| Scpdo EMSA79bp-r | ACAAAGAACACGGCACGCTCTCCTTCCGGAATTACCCCCTGGGGTATCTCTTCCGGCCACCGTAACAGATATACCCAGG |  |
| hrdB EMSA79bp-f | cattcccaagccggtggtcggcccctgtccgccgtggacggggccggaagccgtttttcaacgttccgagaggttgttc | 79bp fragment for EMSA |
| hrdB EMSA79bp-r | gaacaacctctcggaacgttgaaaaacggcttccggccccgtccacggcggacaggggccgaccaccggcttgggaatg |  |
| Scpdo Race Outer | ACGAAGTGTGGTGCGGGGTGT | 5'-RLM-RACE Gene-Specific Primers |
| Scpdo Race inner | CGTAGTCGTTGTGGACGTGGGTC |  |

**References of Table S1 and S2**

Aparicio, J.F., Fouces, R., Mendes, M.V., Olivera, N., and Martin, J.F. (2000) A complex multienzyme system encoded by five polyketide synthase genes is involved in the biosynthesis of the 26-membered polyene macrolide pimaricin in *Streptomyces natalensis*. *Chem Biol* **7**: 895-905.

Cundliffe, E., Bate, N., Butler, A., Fish, S., Gandecha, A., and Merson-Davies, L. (2001) The tylosin-biosynthetic genes of *Streptomyces fradiae*. *Antonie van Leeuwenhoek* **79**: 229-234.

Dulmage, H.T. (1953) The production of neomycin by *Streptomyces fradiae* in synthetic media. *Appl Microbiol* **1**: 103-106.

Elsayed, E.A., Farid, M.A., and El-Enshasy, H.A. (2019) Enhanced Natamycin production by *Streptomyces natalensis* in shake-flasks and stirred tank bioreactor under batch and fed-batch conditions. *BMC Biotechnol* **19**: 46.

Gregory, M.A., Till, R., and Smith, M.C. (2003) Integration site for *Streptomyces* phage phiBT1 and development of site-specific integrating vectors. *J Bacteriol* **185**: 5320-5323.

He, Y., Wang, Z., Bai, L., Liang, J., Zhou, X., and Deng, Z. (2010) Two pHZ1358-derivative vectors for efficient gene knockout in streptomyces. *J Microbiol Biotechnol* **20**: 678-682.

Huang, H., Zheng, G., Jiang, W., Hu, H., and Lu, Y. (2015) One-step high-efficiency CRISPR/Cas9-mediated genome editing in *Streptomyces*. *Acta Biochim Biophys Sin (Shanghai)* **47**: 231-243.

Imai, Y., Sato, S., Tanaka, Y., Ochi, K., and Hosaka, T. (2015) Lincomycin at Subinhibitory Concentrations Potentiates Secondary Metabolite Production by Streptomyces spp. *Appl Environ Microbiol* **81**: 3869-3879.

Jia, N., Ding, M.Z., Luo, H., Gao, F., and Yuan, Y.J. (2017) Complete genome sequencing and antibiotics biosynthesis pathways analysis of *Streptomyces lydicus* 103. *Sci Rep* **7**: 44786.

Jiang, C., Wang, H., Kang, Q., Liu, J., and Bai, L. (2012) Cloning and characterization of the polyether salinomycin biosynthesis gene cluster of *Streptomyces albus* XM211. *Appl Environ Microbiol* **78**: 994-1003.

Kharel, M.K., Subba, B., Basnet, D.B., Woo, J.S., Lee, H.C., Liou, K., and Sohng, J.K. (2004) A gene cluster for biosynthesis of kanamycin from *Streptomyces kanamyceticus*: comparison with gentamicin biosynthetic gene cluster. *Arch Biochem Biophys* **429**: 204-214.

Kieser, T., Bibb, M.J., Buttner, M.J., Chater, K.F., and Hopwood, D.A. (2000) *Practical streptomyces genetics*: John Innes Foundation Norwich.

Mast, Y., Guezguez, J., Handel, F., and Schinko, E. (2015) A Complex Signaling Cascade Governs Pristinamycin Biosynthesis in *Streptomyces pristinaespiralis*. *Appl Environ Microbiol* **81**: 6621-6636.

Matsuoka, M. (1960) Biological studies on antifungal substances produced by *Streptomyces fungicidicus*. *J Antibiot (Tokyo)* **13**: 121-124.

McLean, T.C., Hoskisson, P.A., and Seipke, R.F. (2016) Coordinate Regulation of Antimycin and Candicidin Biosynthesis. *mSphere* **1**.

Mendez, C., Kunzel, E., Lipata, F., Lombo, F., Cotham, W., Walla, M., et al. (2002) Oviedomycin, an unusual angucyclinone encoded by genes of the oleandomycin-producer *Streptomyces antibioticus* ATCC11891. *J Nat Prod* **65**: 779-782.

Mindlin, S.Z., Alikhanian, S.I., Vladimirov, A.V., and Mikhailova, G.R. (1961) A new hybrid strain of an oxytetracycline-producing organism, *Streptomyces rimosus*. *Appl Microbiol* **9**: 349-353.

Olano, C., Garcia, I., Gonzalez, A., Rodriguez, M., Rozas, D., Rubio, J., et al. (2014) Activation and identification of five clusters for secondary metabolites in *Streptomyces albus* J1074. *Microb Biotechnol* **7**: 242-256.

Oliynyk, M., Samborskyy, M., Lester, J.B., Mironenko, T., Scott, N., Dickens, S., et al. (2007) Complete genome sequence of the erythromycin-producing bacterium *Saccharopolyspora erythraea* NRRL23338. *Nat Biotechnol* **25**: 447-453.

Pape, H., and Brillinger, G.U. (1973) Metabolic products of microorganisms. 113. Biosynthesis of thymidine diphospho mycarose in a cell-free system from *Streptomyces rimosus*. *Arch Mikrobiol* **88**: 25-35.

Peschke, U., Schmidt, H., Zhang, H.Z., and Piepersberg, W. (1995) Molecular characterization of the lincomycin-production gene cluster of *Streptomyces lincolnensis* 78-11. *Mol Microbiol* **16**: 1137-1156.

Sui, Q., Liu, W., Lu, C., Liu, T., Qiu, J., and Liu, X. (2009) Extraction and structural identification of the antifungal metabolite of *Streptomyces lydicus* A02. *Sheng wu gong cheng xue bao = Chinese journal of biotechnology* **25**: 840-846.

Takano, H., Toriumi, N., Hirata, M., Amano, T., Ohya, T., Shimada, R., et al. (2016) An ABC transporter involved in the control of streptomycin production in *Streptomyces griseus*. *FEMS Microbiol Lett* **363**.

Veiga, M., Traba, M.P., and Fabregas, J. (1983) Tetrafungin, a new polyene macrolide antibiotic. II. Taxonomy of the producing organism and comparison with nystatin by means of high performance liquid chromatography. *J Antibiot (Tokyo)* **36**: 776-783.

Wang, F., Fu, S.N., Bao, Y.X., Yang, Y., Shen, H.F., Lin, B.R., and Zhou, G.X. (2017) Kitamycin C, a new antimycin-type antibiotic from *Streptomyces antibioticus* strain 200-09. *Nat Prod Res* **31**: 1819-1824.

Wang, X.J., Yan, Y.J., Zhang, B., An, J., Wang, J.J., Tian, J., et al. (2010) Genome sequence of the milbemycin-producing bacterium *Streptomyces bingchenggensis*. *J Bacteriol* **192**: 4526-4527.

Xia, Y., Lu, C., Hou, N., Xin, Y., Liu, J., Liu, H., and Xun, L. (2017) Sulfide production and oxidation by heterotrophic bacteria under aerobic conditions. *ISME J* **11**: 2754-2766.

Yin, S., Wang, W., Wang, X., Zhu, Y., Jia, X., Li, S., et al. (2015) Identification of a cluster-situated activator of oxytetracycline biosynthesis and manipulation of its expression for improved oxytetracycline production in *Streptomyces rimosus*. *Microb Cell Fact* **14**: 46.

Yu, Z., Zhu, H., Dang, F., Zhang, W., Qin, Z., Yang, S., et al. (2012) Differential regulation of antibiotic biosynthesis by DraR-K, a novel two-component system in *Streptomyces coelicolor*. *Mol Microbiol* **85**: 535-556.

Zhang, J., An, J., Wang, J.J., Yan, Y.J., He, H.R., Wang, X.J., and Xiang, W.S. (2013) Genetic engineering of *Streptomyces bingchenggensis* to produce milbemycins A3/A4 as main components and eliminate the biosynthesis of nanchangmycin. *Appl Microbiol Biotechnol* **97**: 10091-10101.

Zhou, T.C., Kim, B.G., and Zhong, J.J. (2014) Enhanced production of validamycin A in *Streptomyces hygroscopicus* 5008 by engineering validamycin biosynthetic gene cluster. *Appl Microbiol Biotechnol* **98**: 7911-7922.
